# The *Cdkn2a* gene product p19 alternative reading frame (p19ARF) is a critical regulator of IFNβ-mediated Lyme arthritis

**DOI:** 10.1371/journal.ppat.1010365

**Published:** 2022-03-24

**Authors:** Jinze Li, Ying Ma, Jackie K. Paquette, Amanda C. Richards, Matthew A. Mulvey, James F. Zachary, Cory Teuscher, Janis J. Weis

**Affiliations:** 1 Department of Pathology, University of Utah, Salt Lake City, Utah, United States of America; 2 Department of Veterinary Pathobiology, University of Illinois at Urbana-Champaign, Urbana, Illinois, United States of America; 3 Department of Medicine, Vermont Center for Immunology and Infectious Diseases, Larner College of Medicine, The University of Vermont, Burlington, Vermont, United States of America; University of Toronto, CANADA

## Abstract

Type I interferon (IFN) has been identified in patients with Lyme disease, and its abundant expression in joint tissues of C3H mice precedes development of Lyme arthritis. Forward genetics using C3H mice with severe Lyme arthritis and C57BL/6 (B6) mice with mild Lyme arthritis identified the *Borrelia burgdorferi arthritis-associated locus 1* (*Bbaa1*) on chromosome 4 (Chr4) as a regulator of *B*. *burgdorferi*-induced IFNβ expression and Lyme arthritis severity. B6 mice introgressed with the C3H allele for *Bbaa1* (B6.C3-*Bbaa1* mice) displayed increased severity of arthritis, which is initiated by myeloid lineage cells in joints. Using advanced congenic lines, the physical size of the *Bbaa1* interval has been reduced to 2 Mbp, allowing for identification of potential genetic regulators. Small interfering RNA (siRNA)-mediated silencing identified *Cdkn2a* as the gene responsible for *Bbaa1* allele-regulated induction of IFNβ and IFN-stimulated genes (ISGs) in bone marrow-derived macrophages (BMDMs). The *Cdkn2a*-encoded p19 alternative reading frame (p19ARF) protein regulates IFNβ induction in BMDMs as shown by siRNA silencing and overexpression of ARF. *In vivo* studies demonstrated that p19ARF contributes to joint-specific induction of IFNβ and arthritis severity in *B*. *burgdorferi*-infected mice. p19ARF regulates *B*. *burgdorferi*-induced IFNβ in BMDMs by stabilizing the tumor suppressor p53 and sequestering the transcriptional repressor BCL6. Our findings link p19ARF regulation of p53 and BCL6 to the severity of IFNβ-induced Lyme arthritis *in vivo* and indicate potential novel roles for p19ARF, p53, and BCL6 in Lyme disease and other IFN hyperproduction syndromes.

## Author summary

Lyme disease is caused by infection with the tick-transmitted bacterium *Borrelia burgdorferi*. Although different isolates of *B*. *burgdorferi* have distinct potential for dissemination and tissue invasion, factors intrinsic to the infected host also play an important role in directing the severity of Lyme disease. In the animal model, infected C3H mice develop severe Lyme arthritis following elevation of type I IFN in joint tissue, while in C57BL/6 (B6) mice arthritis is mild and not associated with type I IFN. We demonstrated that the *Borrelia burgdorferi arthritis-associated locus 1* (*Bbaa1*) on chromosome 4 (Chr4) intrinsically controls the magnitude of IFNβ production and the severity of Lyme arthritis in C3H vs B6 mice. The *Cdkn2a* gene was positionally identified as the regulator of IFNβ within *Bbaa1*, and determined to function through its protein product p19 alternative reading frame (p19ARF). ARF regulates IFNβ expression and Lyme arthritis severity by modulating the activities of the tumor suppressor p53 and transcriptional repressor BCL6. Our study provides new insight and potential therapeutic targets for the investigation of type I IFN-dependent Lyme arthritis and other IFN-driven diseases.

## Introduction

Lyme disease is caused by infection with the tick-transmitted spirochete *Borrelia burgdorferi* [1], and with 476,000 cases per year, it is the most common vector-borne disease in the United States [2]. Patients with Lyme disease display a spectrum of disease symptoms and severity [3] ranging from erythema migrans at the site of the tick bite to disseminated symptoms, including peripheral neuropathies, meningitis/encephalitis, carditis, and arthritis [1]. Lyme arthritis occurs in about 27.5% of untreated patients and is often characterized by synovitis in the knee joints [1,3,4]. Although the acute Lyme disease symptoms can usually be treated with appropriate antibiotic treatment [5,6], 10–20% of the patients continue to display symptoms despite antibiotic treatment, referred to as posttreatment Lyme disease syndrome [3,7,8]. The range in clinical manifestation is partially determined by the genetic properties of the infecting isolate of *B*. *burgdorferi*; some isolates are restricted to cutaneous sites while other isolates disseminate from the skin to other organs and tissues in patients and experimental animals [9–12]. Inherent distinctions in host responses also contribute to the range of symptoms, organs involved, and speed of recovery [13–15].

In 1990, Barthold and colleagues established unequivocally that host genetics is a major determinant of disease severity by infecting several inbred strains of mice with a single isolate of *B*. *burgdorferi*; they found that C3H mice displayed severe arthritis and carditis, C57BL/6 (B6) mice displayed mild disease, and the other strains of mice displayed intermediate symptoms [14]. Subsequent studies established that differences in arthritis severity did not depend on the level of *B*. *burgdorferi* in joint tissues or the MHC haplotype [16–18]. Studies in patients have revealed the contribution of both inflammatory and innate defenses to disease severity and that MHC linkage plays a role in chronic, but not acute, Lyme disease [19]. Using forward genetics between C3H and B6 mice, we identified six quantitative trait loci (QTL) on five mouse chromosomes that regulate Lyme arthritis severity, termed *Borrelia burgdorferi arthritis-associated (Bbaa)* loci [20,21]. Previously, we identified beta-glucuronidase (GUSB) on mouse chromosome 5 (Chr5) as a major regulator of *B*. *burgdorferi* arthritis-associated locus 2 (*Bbaa2*) [22]. A second highly penetrant QTL, *B*. *burgdorferi associated arthritis locus 1* (*Bbaa1*), maps to chromosome 4 (Chr4) and includes the type I interferon (IFN) gene cluster [20]. This was of great interest because robust induction of a type I IFN signature response was identified in C3H mice and linked to Lyme arthritis through use of a type I IFN receptor blocking antibody and by ablation of the type I IFN receptor gene (IFNAR1) [17,23–25]. This observation was followed by development of B6.C3-*Bbaa1* congenic mice in which the C3H allele of *Bbaa1* was introgressed onto B6 mice [26]. These congenic mice demonstrated that *Bbaa1* regulates arthritis severity by upregulating IFNβ [26,27]. Importantly, type I IFN did not play a role in controlling the number of *B*. *burgdorferi* spirochetes in tissues or in the development of antibody responses to *B*. *burgdorferi* [17]. Surprisingly, the skeletal muscle regulatory protein myostatin (MSTN) was identified as a novel downstream mediator that links IFNβ to severe arthritis in response to *B*. *burgdorferi* infection [27].

The absence of single-nucleotide polymorphisms (SNPs) when comparing the C3H and B6 IFNβ genes indicated that additional factors within C3H *Bbaa1* allele are responsible for the greater expression of IFNβ and Lyme arthritis severity. We have shown, by positional cloning, that the protein encoded by the p19 alternative reading frame (p19ARF, referred to as ARF) of the tumor suppressor gene *Cdkn2a* regulates IFNβ in myeloid cells, which are responsible for the initiation of the IFN profile in joint tissues [25]. We have also shown that suppression and blocking of the ARF-regulated proteins p53 and BCL6 in macrophages leads to altered expression of type I IFN, and blocking of BCL6 in B6 mice induces IFNβ activation in joint tissues, thereby increasing the severity of Lyme arthritis. This mechanistic study reveals the pathways involved in *Cdkn2a*-regulated expression of IFNβ and development of severe Lyme arthritis. Identification of genetic factors that act upstream of type I IFN could lead to development of new therapeutic targets for the treatment of type I IFN-dependent Lyme arthritis and offer support for the investigation of other IFN-driven diseases.

## Results

### Identification of Cdkn2a as a potential regulator of IFNβ expression

Using a forward genetics approach, we determined previously that the *Bbaa1* locus on Chr4 intrinsically controls IFNβ production, and, through the development of B6.C3-*Bbaa1*congenic mice we determined that this locus is one of the major genetic regulators of severe Lyme arthritis [27] (S1A Fig). To identify and localize the genetic elements that regulate IFNβ within this region of Chr4, we further back-crossed B6.C3-*Bbaa1* to B6 mice to reduce the physical contribution of C3H genes and to develop interval-specific recombinant congenic lines (ISRCLs). These congenic mice revealed the region surrounding the *Ifnb* gene that primarily contributes to Lyme arthritis development (S1B Fig). A newly developed congenic mouse, ISRCL5, greatly reduced the physical region associated with penetrant arthritis to 2.2 Mbps (S1B Fig). Thus, genetic factors that regulate IFNβ production and arthritis severity were located within the physical boundary of the ISRCL5 region. RNA-seq analysis of BMDMs from wild type B6 mice, ISRCL3, and ISRCL4 mice identified genes within the 2.2 Mbps region (S1 Table). Six protein-coding genes and one long intergenic noncoding RNA (lincRNA) within the 2.2 Mbps interval were identified as candidate regulatory genes based on the criteria of 1) being expressed in BMDMs and 2) the presence of SNPs when comparing B6 and C3H mice (Table 1).

**Table 1:** RNA-seq identified 6 candidate genes and 1 lincRNA in the 2 Mbps *ISRCL5* interval.

Gene symbol	SNPs	Gene title	Biotype	Chr4 position
Focad	Y	Focadhesin	Protein coding	88,094,629–88,411,011
Hacd4	Y	3-hydroxyacyl-CoA dehydratase 4	Protein coding	88,396,144–88,438,928
Ifnb1	N	**Interferon beta 1**	**Protein coding**	**88,522,025–88,522,794**
Klhl9	Y	Kelch-like 9	Protein coding	88,718,292–88,722,465
Mtap	Y	Methylthioadenosine phosphorylase	Protein coding	89,137,122–89,181,081
Gm12606	Y	Predicted gene 12606	lincRNA	89,235,699–89,273,403
Cdkn2a	Y	Cyclin-dependent kinase inhibitor 2A	Protein coding	89,274,471–89,294,653
Cdkn2b	Y	Cyclin-dependent kinase inhibitor 2B	Protein coding	89,306,289–89,311,032

The listed genes were expressed in joint tissue and BMDMs and possessed SNPs upon comparison of the genes in C3H and B6 mice.

RNA was prepared from BMDMs from ISRCL3, ISRCL4, and B6 mice before and after stimulation with *B*. *burgdorferi* for 3 or 6 h.

B6: n = 4, ISRCL4: n = 4, ISRCL3: n = 3

**Fig 1:**
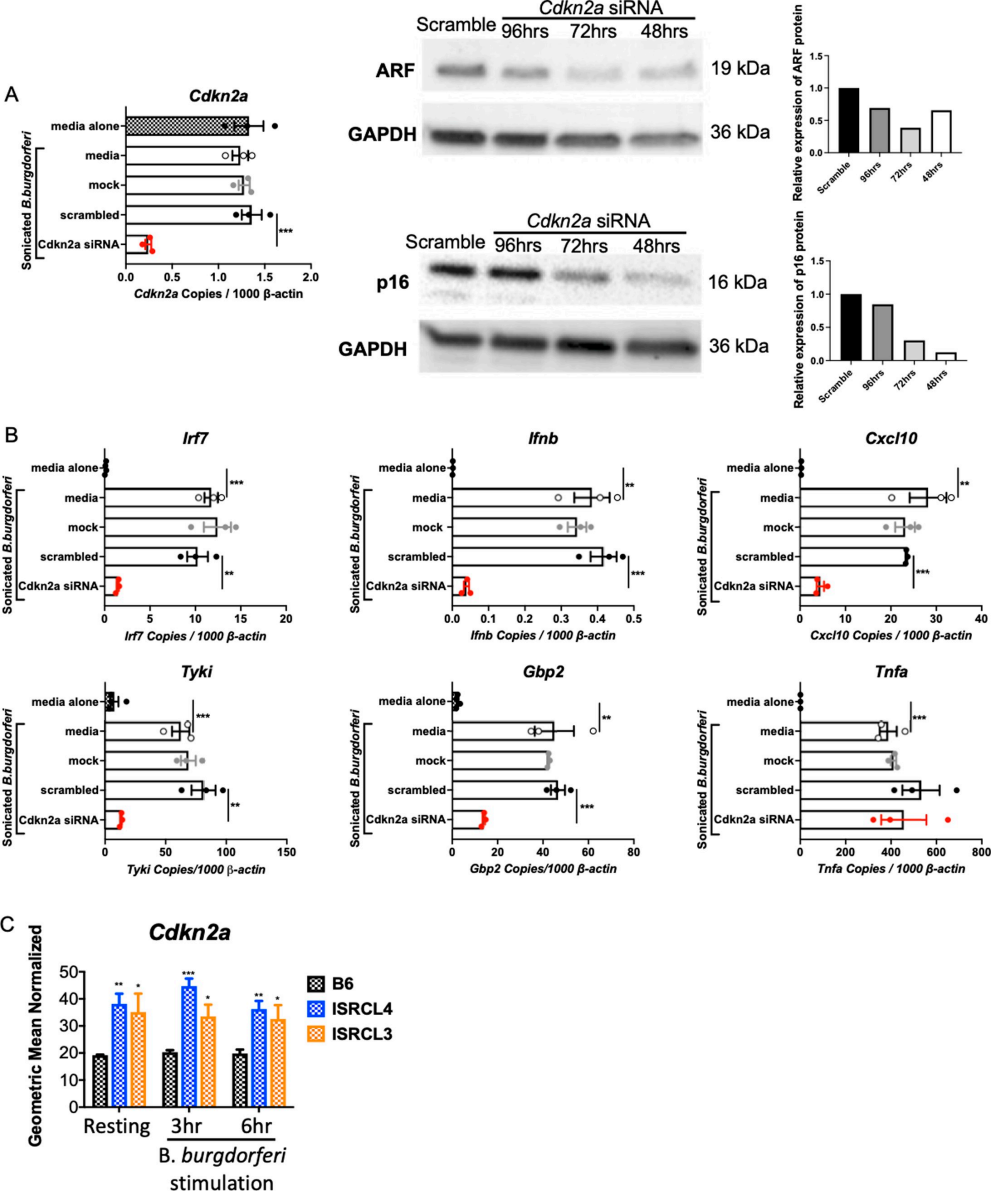
Silencing of *Cdkn2a* suppressed expression of *Ifnb* and ISGs. A) *Cdkn2a* was silenced in B6.C3-*Bbaa1* BMDMs upon transfection of 100 nM *Cdkn2a* Smartpool siRNA using the Neon electroporation system. Scrambled siRNA was transfected as the control. Transfected macrophages were treated with 5 μg/ml sonicated *B*. *burgdorferi* for 6 h prior to collection for RNA and protein extraction. The induction of IFN responses by *B*. *burgdorferi* treatment is confirmed by comparing *B*. *burgdorferi* treated media (no-silencing) and BSK media alone (no stimulus) groups. *Cdkn2a* knockdown efficiency was assessed by qPCR with normalization to *β-actin* and Western blot. The quantification of *Cdkn2a-*encoded ARF and p16 protein levels is shown in the bar graph on the right side of the western blots. Significance was determined by Student *t*-test. Data are shown as mean ± SEM (n = 3 per group). B) The impact of *Cdkn2a* silencing on expression of *Ifnb* and the IFN downstream genes *Cxcl10*, *Tyki*, and *Gbp2* was determined by qPCR normalized to *β-actin*. All transfection experiments were repeated at least twice. Significance was determined by Student *t*-test. Data are shown as mean ± SEM (n = 3 per group). C) RNA-seq data demonstrated that *Cdkn2a* was expressed constitutively at a higher level in BMDMs from ISRCL3 and ISRCL4 mice than in BMDMs from B6 mice following stimulation with live *B*. *burgdorferi* for 3 and 6 h. Significance was assessed by 1-way ANOVA followed by Dunnett’s multiple comparison test versus B6. Error bars indicate SEM (n = 3 or 4 per group). *p < 0.05, **p < 0.01, ***p < 0.001, ****p < 0.0001.

Genes identified in Table 1 were individually silenced by transfecting BMDMs from B6.C3-*Bbaa1* mice with SMARTpool siRNAs, and the impact of the silencing on *B*. *burgdorferi*-induced IFN responses was evaluated. Following treatment with sonicated *B*. *burgdorferi*, expression of IFNβ and downstream interferon inducible gene (ISG) transcripts was measured by qRT-PCR. Silencing of a single gene, *Cdkn2a* (Fig 1A), suppressed expression of *Irf7*, *Ifnb*, and ISGs (Fig 1B). *Cdkn2a* is a cyclin-dependent kinase inhibitor that functions as a tumor suppressor. Importantly, silencing of *Cdkn2a* did not result in suppression of *B*. *burgdorferi*-induced upregulation of *Tnfa*, a transcript for which induction is dependent on parallel activation of a pathway involving MyD88 and NF-κB (Fig 1B). This indicates that silencing of *Cdkn2a* selectively suppresses IFNβ upregulation without generalized suppression of other transcriptional responses. Thus, *Cdkn2a* was determined to be the major regulator of *B*. *burgdorferi*-induced upregulation of IFNβ within the narrowed *Bbaa1* locus.

**Fig 2:**
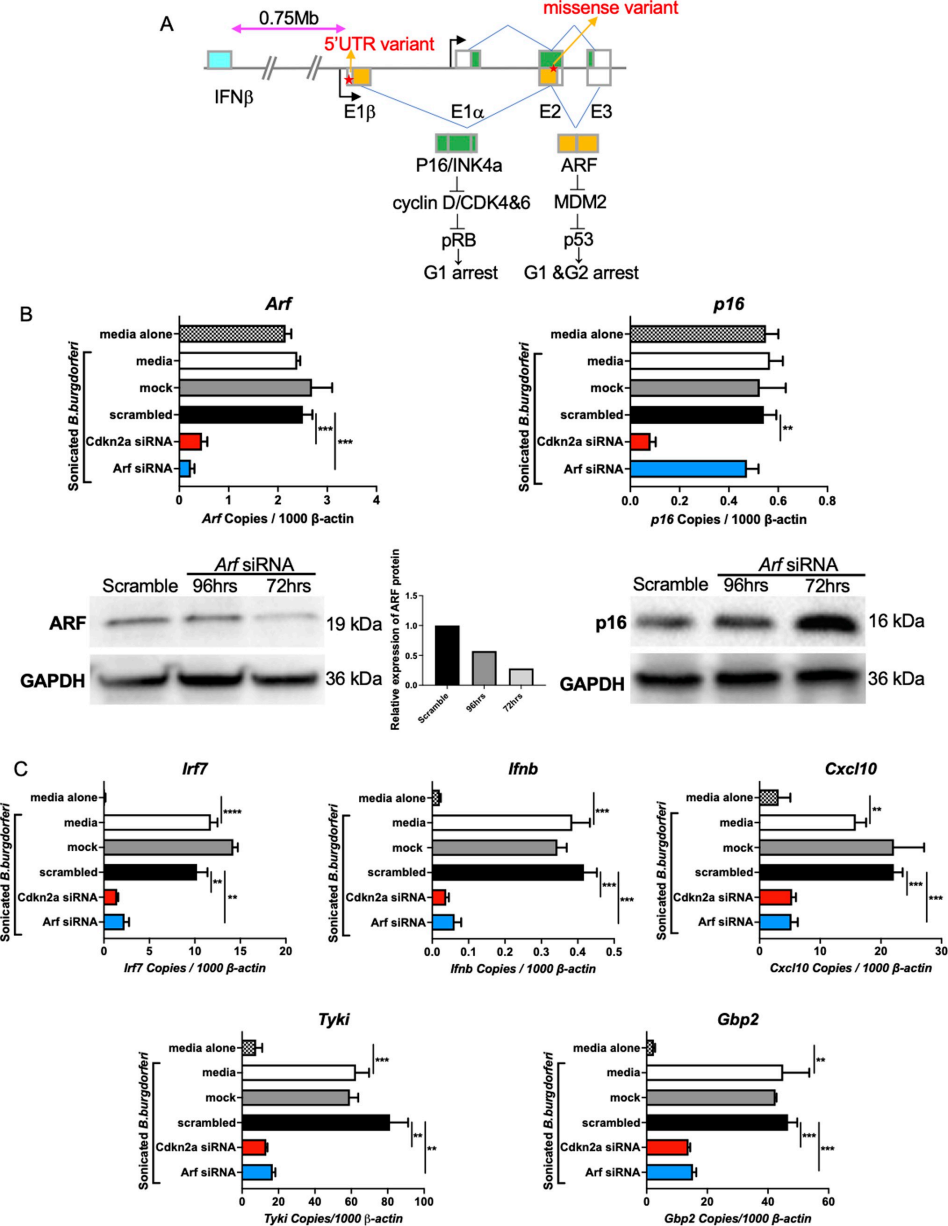
ARF regulates the IFNβ pathway. A) *Cdkn2a* encodes two proteins, p16 and ARF, and possesses 2 SNPs in the transcribed sequences. B) *Arf* expression was silenced with *Arf* siRNA (100 nM) in B6.C3-*Bbaa1* BMDMs. ARF and p16 expression levels were evaluated by qPCR with normalization to *β-actin* and Western blot following treatment with 5 μg/ml sonicated *B*. *burgdorferi* for 6 h. IFN responses induced by *B*. *burgdorferi* treatment are indicated as *B*. *burgdorferi-*treated media, while those not receiving B. burgdorferi are indicated by media alone. Significance was determined by Student *t*-test. Error bars indicate the SEM (n = 3 per group). C) The impact of *Arf* silencing on expression of *B*. *burgdorferi*-stimulated IFN responses in B6.C3-*Bbaa1* BMDMs were determined by qPCR normalized to *β-actin*. Significance of differences was measured by Student *t*-test. Data are shown as mean ± SEM (n = 3 per group). **p < 0.01, ***p < 0.001, ****p < 0.0001.

The *Cdkn2a* gene encodes two proteins, p16 (INK4a) and ARF, that incorporate Exon 2, but with distinct reading frames [28]. Both proteins have been studied extensively due to their effects on the cell cycle via distinct pathways. *P16* and *Arf* are transcribed from separated promoters and they have unique first exons, E1α and E1β, but share exons 2 and 3 (E2 and E3) [28]. The sequences that encode p16 are shown in green and the sequences that encode ARF are shown in orange (Fig 2A). Two SNPs have been identified by comparing the C3H and B6 mouse *Cdkn2a* gene sequences. The first SNP is within E2 and is a missense variant that alters the coding sequence and, thus, could alter both p16 and ARF protein function. The second SNP is in the 5’UTR of E1β of ARF and could affect ARF expression. Interestingly, analysis of RNA-seq data from BMDMs revealed that *Cdkn2a* is constitutively expressed at a higher level in BMDMs from ISRCL3 and ISRCL4 congenic mice than from B6 mice, and this difference is maintained following treatment with *B*. *burgdorferi* (Fig 1C). The differential expression of ARF was confirmed at the RNA and protein level with BMDMs from B6.C3-*Bbaa1* congenic mice and B6 mice (S2A and S2B Fig). These results suggest that the 5’UTR SNP upregulates IFNβ via enhanced expression of ARF RNA and protein.

### ARF regulates IFNβ expression

**Fig 3:**
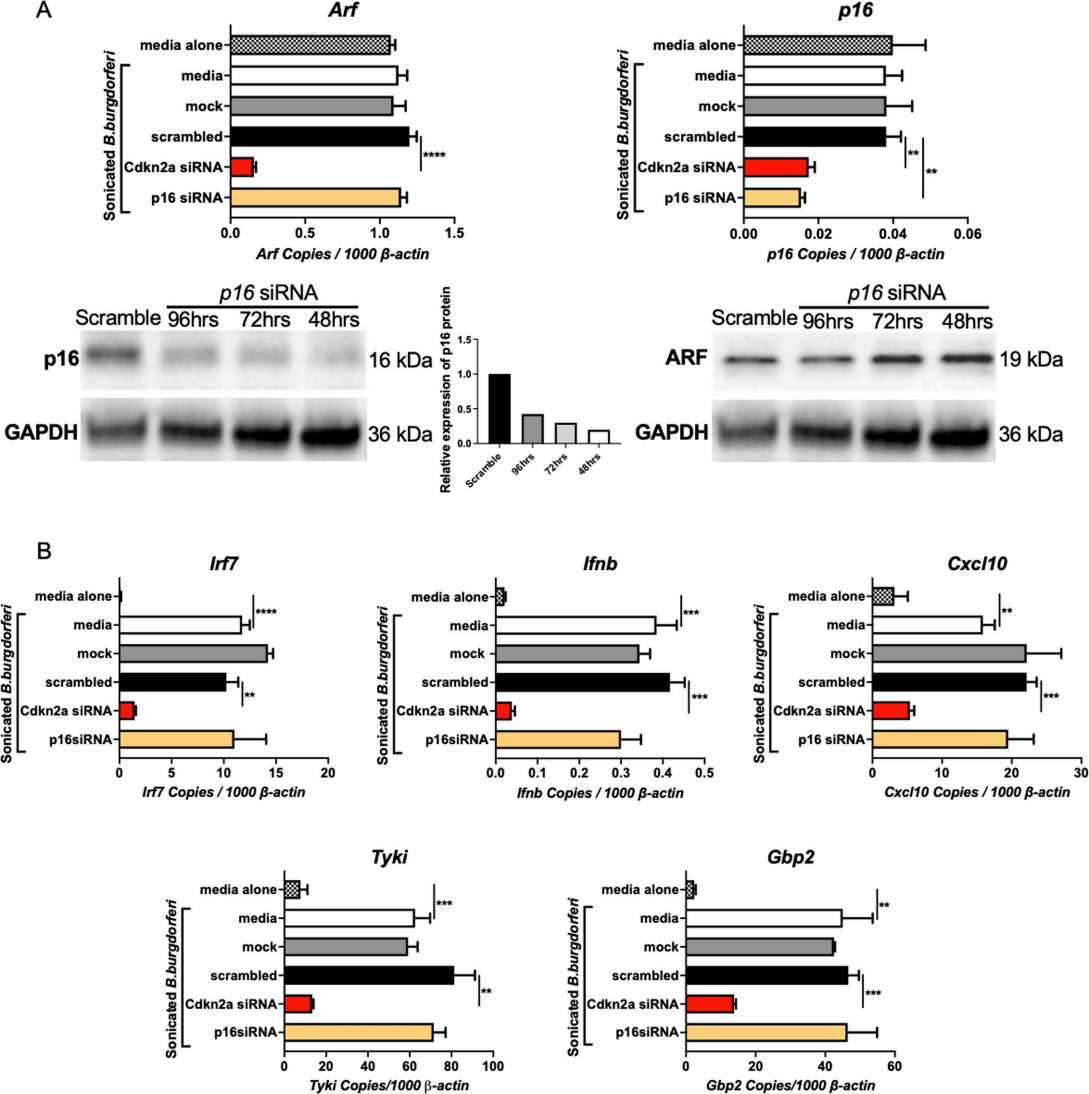
IFNβ modulating activity of *Cdkn2a* is not due to p16 protein. A) Transfection of *p16* siRNA (100 nM) into B6.C3-*Bbaa1* BMDMs silenced *p16*. ARF and p16 expression levels were evaluated by qPCR with normalization to *β-actin* and Western blot following treatment with 5 μg/ml sonicated *B*. *burgdorferi* for 6 h. Significance was determined by Student *t*-test. Data are shown as mean ± SEM (n = 3 per group). B) The impact of selective silencing of *p16* on expression of *B*. *burgdorferi*-stimulated *Ifnb* and the IFN downstream genes *Cxcl10*, *Tyki*, and *Gbp2* in B6.C3-*Bbaa1* BMDMs was determined by qPCR normalized to *β-actin*. Error bars indicate SEM (n = 3 per group). **p < 0.01, ***p < 0.001, ****p < 0.0001.

**Fig 4:**
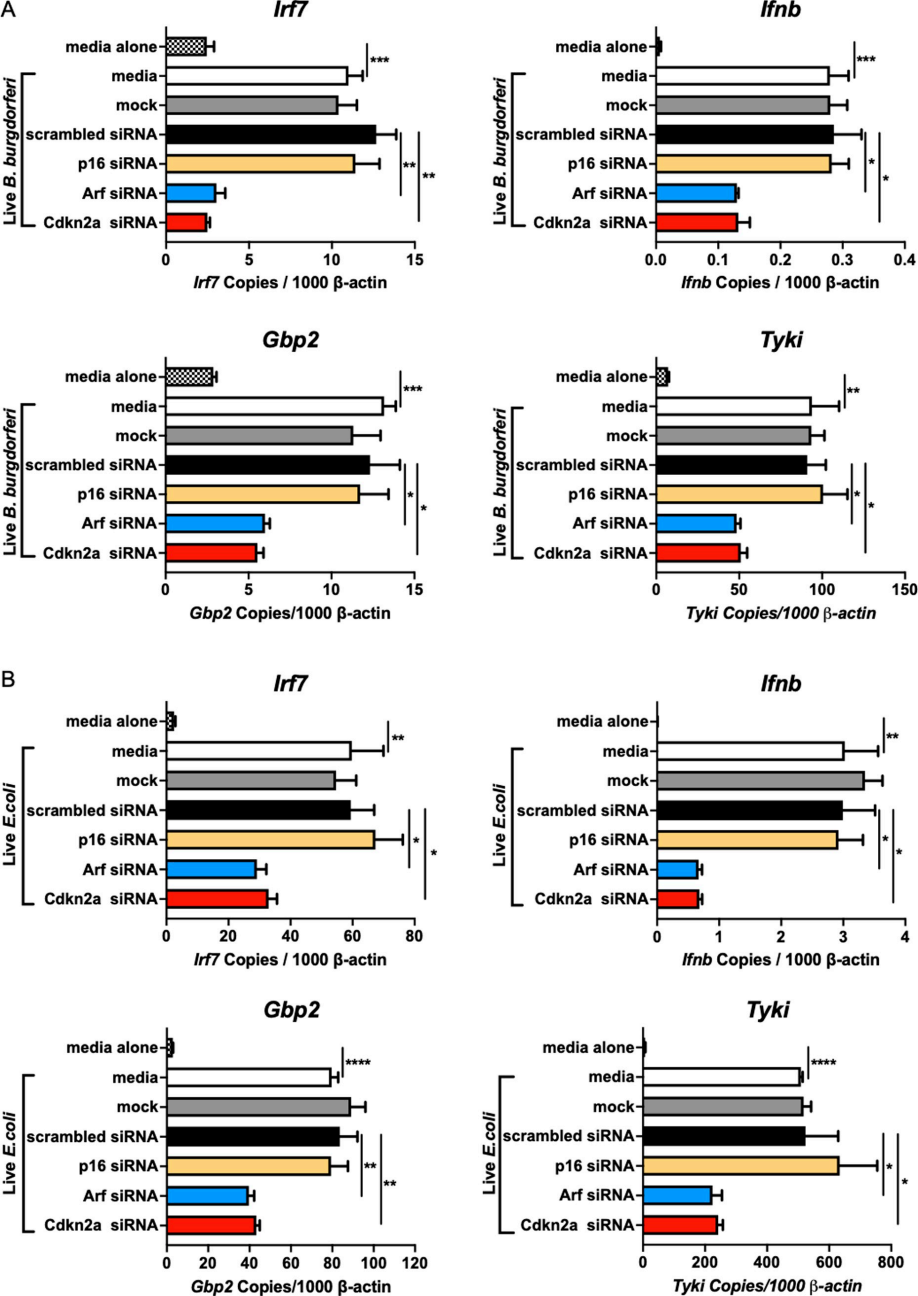
ARF impacts the magnitude of IFNβ expression in response to living *B*. *burgdorferi* and pathogenic *E*.*coli*. A) The impact of *Cdkn2a*, *Arf* and *p16* silencing on IFN expression in response to 7.4×10^6^ live *B*. *burgdorferi* (MOI of 10) for 6 h is shown. Data are shown as mean ± SEM (n = 3 per group). B) The impact of silencing of *Cdkn2a*, *Arf* and *p16* on live *E*. *coli* CFT073 stimulated IFN responses was determined by qPCR normalized to *β-actin* following stimulation for 6 h, at an MOI of 10. Error bars indicate SEM (n = 3 per group). Significance of the differences was measured by Student *t*-test. *p < 0.05, **p < 0.01, ***p < 0.001, ****p < 0.0001.

To study the mechanism of *Cdkn2a* modulation of IFN production, it was necessary to discriminate between the effects of the two encoded proteins, ARF and p16. Therefore, ARF and p16 were silenced individually using siRNAs and the effect on IFNβ induction assessed. qPCR and Western blot analysis showed that the *Arf*-specific siRNA that targeted E1β specifically silenced *Arf*, but not *p16* (Fig 2B). The selective silencing of *Arf* resulted in a 50–80% reduction in *Irf7*, *Ifnb*, and IFN gene expression, which is similar to the level of suppression obtained upon silencing with the *Cdkn2a* SMARTpool siRNA (Fig 2C). *P16* was silenced with an siRNA that targeted the E1α exon, which is unique to p16, and this siRNA silenced p16 RNA and protein, but not ARF (Fig 3A). In contrast to the silencing of *Arf*, the silencing of *p16* did not result in suppression of *Irf7*, *Ifnb*, or IFN gene expression following treatment with *B*. *burgdorferi* (Fig 3B). These results showed that the IFNβ modulating activity of *Cdkn2a* is exclusively due to the ARF protein with no contribution from p16. Additional experiments performed with an equivalent number of live *B*. *burgdorferi* gave similar results to those using sonicated bacteria, indicating ARF enhances IFNβ induction by intact cultured organisms (Fig 4A). Interestingly, ARF also impacts the magnitude of IFNβ expression in response to the *Escherichia coli* (*E*. *coli*) ExPEC reference strain CFT073 [29] (Fig 4B), demonstrating the involvement of ARF in responding to other human pathogens.

**Fig 5:**
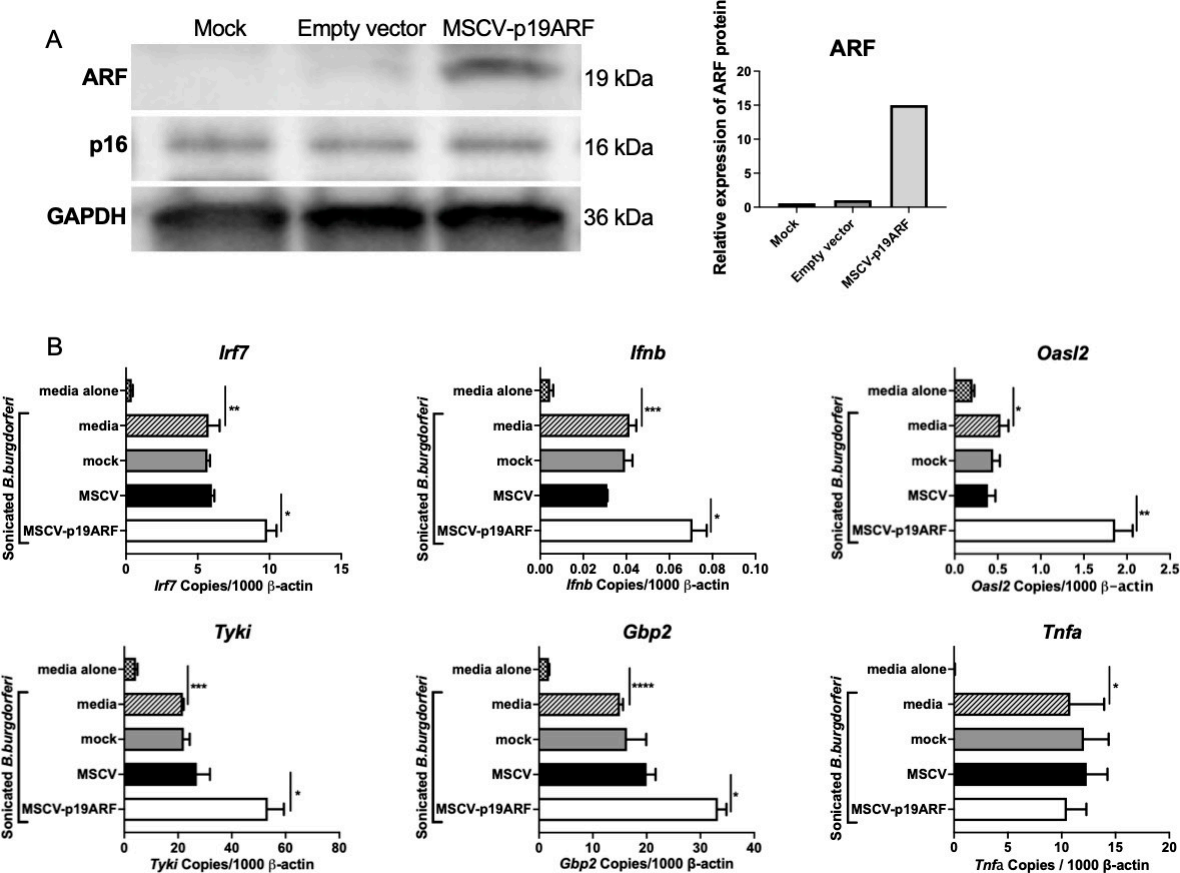
Overexpression of ARF in B6 macrophages enhances *Ifnb* expression. A) ARF was overexpressed in B6 BMDMs by transfection of the MSCV-p19ARF plasmid. The MSCV vector was transfected as the control. The transfected cells were allowed to rest for 72 h before the addition of sonicated *B*. *burgdorferi* for 6 h. The induction of IFN responses by *B*. *burgdorferi* treatment is confirmed by comparing the data from *B*. *burgdorferi* treated media with media alone. Total RNA and protein were isolated for analysis. B) The impact of ARF overexpression on activation of *Ifnb* and ISGs in response to sonicated *B*. *burgdorferi* stimulation in B6 BMDMs was determined by qPCR normalized to *β-actin*. Significance was calculated by Student *t*-test. Error bars indicate SEM (n = 3 per group). *p < 0.05, **p < 0.01.

Because we hypothesized that the 5’SNP found in the C3H allele of ARF was an expression-level polymorphism, we tested whether overexpression of the B6 allele of ARF in B6 BMDMs would result in heightened expression of IFNβ similar to the C3H allele. The *Arf* plasmid consists of a murine stem cell virus (MSCV) promoter and an internal ribosome entry site (IRES)-driven green fluorescent protein (GFP) gene downstream of the B6 allele of *Arf*. The Neon electroporation system was used to transfect BMDMs from B6 mice with the MSCV-p19ARF plasmid. At 72 h after transfection, ARF protein production was about 15-fold greater than the endogenous level while p16 expression was unaltered (Fig 5A). The effect of exogenous ARF on the IFN response was determined after treating the transfected cells with sonicated *B*. *burgdorferi*. Exogenous expression of ARF in B6 BMDMs resulted in increased expression of *Ifnb*, *Irf7*, and ISGs in response to *B*. *burgdorferi* (Fig 5B), which is similar to the observations of *B*. *burgdorferi*-treated B6.C3-*Bbaa1* BMDMs [27]. Transfection of the empty MSCV vector did not affect expression of IFNβ and ISGs. These results support the conclusion that ARF regulates IFNβ expression.

### Multiple microbial PAMPs utilize ARF in the upregulation of myeloid IFNβ

*B*. *burgdorferi* has been demonstrated to stimulate IFN-responsive genes in joint tissues of infected mice and in tissues and blood of Lyme disease patients [17,30–32]. Multiple *B*. *burgdorferi* ligands contribute to induction of the type I IFN response in cultured human and murine macrophages/monocytes, including *B*. *burgdorferi* RNA, lipoproteins, glycolipids, secreted molecules, and peptidoglycan [33–38]. To identify the responses to pathogen-associated molecular patterns (PAMPs) that could be influenced by ARF, a number of ligands were tested with ARF silenced B6.C3-*Bbaa1* BMDMs (Table 2). *B*. *burgdorferi* PAMPs, including lipopeptide Pam_3_Cys, *B*. *burgdorferi* RNA and muramyl dipeptide (MDP), and non-*Borrelia* PAMPs poly (I:C) and lipopolysaccharide (LPS) were tested for their influences on IFNβ responses in ARF or p16 silenced BMDMs [33–44].

ARF silencing in cells treated with sonicated *B*. *burgdorferi* resulted in reduced induction of *Ifnb* and *ISGs* (Table 2). Similarly, stimulation of the IFN response by *B*. *burgdorferi* RNA was regulated by ARF expression (Table 2), a pathway involving toll-like receptor 7 (TLR7) and TLR8 and their downstream effectors [37,44]. Lipoproteins bind to TLR2 and signal through the MyD88/MAL adapter pathway [42]. IFN induction by the synthetic lipopeptide Pam_3_Cys was also dependent on ARF expression (Table 2). These findings offer clues to the mechanism of ARF regulation of the IFNβ pathway and may facilitate the discovery of intermediary proteins involved in ARF regulation of IFNβ. Although *B*. *burgdorferi* does not follow the Gram +/- staining convention, it does possess peptidoglycan with the MDP component, which is sensed intracellularly by NOD2 [39,40]. Peptidoglycan has been implicated in murine Lyme arthritis and in late-stage Lyme arthritis in patients [34], thus it is important to note that induction of *Ifnb* and ISGs transcripts by MDP is independent of ARF (Table 2).

LPS is a classically studied bacterial PAMP that uses TLR4 recognition and the MyD88 and TRIF dependent pathways for signaling [41,43]. Although *B*. *burgdorferi* lacks LPS, its importance in severe inflammatory responses prompted us to test the impact of ARF suppression on *E*. *coli* LPS-induced *Ifnb*. In fact, the IFN response to LPS was partially reduced by the selective silencing of ARF (Table 2). An additional PAMP not found in *B*. *burgdorferi*, poly(I:C), is a synthetic mimetic of viral dsRNA that strongly induces type I IFN stimulation through TLR3 [33,45]. Importantly, IFNβ stimulation by poly(I:C) was not influenced by differential expression of ARF (Table 2), which questions the involvement of the ARF-IFNβ pathway in the universal signaling response to viral infections. p16 silencing had no effect on IFN responses induced by PAMPs associated with *B*. *burgdorferi* or *E*.*coli* (Figs 3 and 4), which is consistent with the exclusive role of ARF in regulating IFNβ.

**Table 2:** Multiple PAMPs induce ARF-regulated upregulation of the IFN response.

IFN inducible genes					
Treatment	Transfection	*Ifnb*	*Tyki*	*Gbp2*	*Iigp*
Medium	Scrambled siRNA	^a^0.01 ± 0.00	3.81 ± 0.81	1.88 ± 0.32	0.50 ± 0.11
	*Arf* siRNA	0.01 ± 0.00	7.02 ± 0.80	2.63 ± 0.17	0.66 ± 0.06
*B*. *burgdorferi*	Scrambled siRNA	0.42 ± 0.04	81.34 ± 9.87	46.61 ± 3.05	1.79 ± 0.14
	*Arf* siRNA	^b^ **0.06 ± 0.02**	**16.84 ± 1.42**	**15.27 ± 1.09**	**0.65 ± 0.13**
Bb RNA	Scrambled siRNA	0.31 ± 0.01	33.55 ± 2.41	41.71 ± 1.93	4.02 ± 0.35
	*Arf* siRNA	**0.07 ± 0.01**	**8.08 ± 0.55**	**15.50 ± 0.87**	**1.00 ± 0.04**
Pam3Cys	Scrambled siRNA	0.23 ± 0.03	34.11 ± 1.65	34.24 ± 2.16	1.24 ± 0.23
	*Arf* siRNA	**0.08 ± 0.01**	**9.90 ± 0.77**	**18.28 ± 1.36**	**0.33 ± 0.02**
MDP	Scrambled siRNA	0.01 ± 0.00	1.50 ± 0.12	6.49 ± 5.93	0.77 ± 0.69
	*Arf* siRNA	0.00 ± 0.00	5.42 ± 0.62	2.71 ± 0.08	0.55 ± 0.06
LPS	Scrambled siRNA	0.80 ± 0.05	264.05 ± 30.36	103.43 ± 3.60	10.41 ± 1.11
	*Arf* siRNA	**0.39 ± 0.01**	**148.52 ± 16.58**	**70.50 ± 6.10**	**5.28 ± 0.84**
Poly I:C	Scrambled siRNA	0.50 ± 0.05	133.66 ± 15.98	72.97 ± 4.02	16.34 ± 1.51
	*Arf* siRNA	0.53 ± 0.14	129.15 ± 17.23	66.62 ± 2.97	14.27 ± 1.27

B6.C3-*Bbaa1* BMDMs were transfected with *Arf* or scrambled siRNAs using the Neon electroporation system. After 48 h of recovery, the transfected macrophages were treated with 5 μg/ml sonicated *B*. *burgdorferi*, 2 μg/ml *B*. *burgdorferi* RNA, 200 ng/ml lipopeptide Pam3Cys, 10 μg/ml MDP, 100 ng/ml LPS, or poly I:C (10 ng/ml or 20 μg/ml) for 6 h prior to sample collection. Transcripts were assessed by qPCR.

^a^Values represent mean ± SEM.

^b^Numbers in bold indicate a significant decrease in induction of the indicated transcript following *Arf* silencing compared with cells receiving scrambled siRNA. *p* < 0.05.

### ARF expression modulates the severity of Lyme arthritis in mice

**Fig 6:**
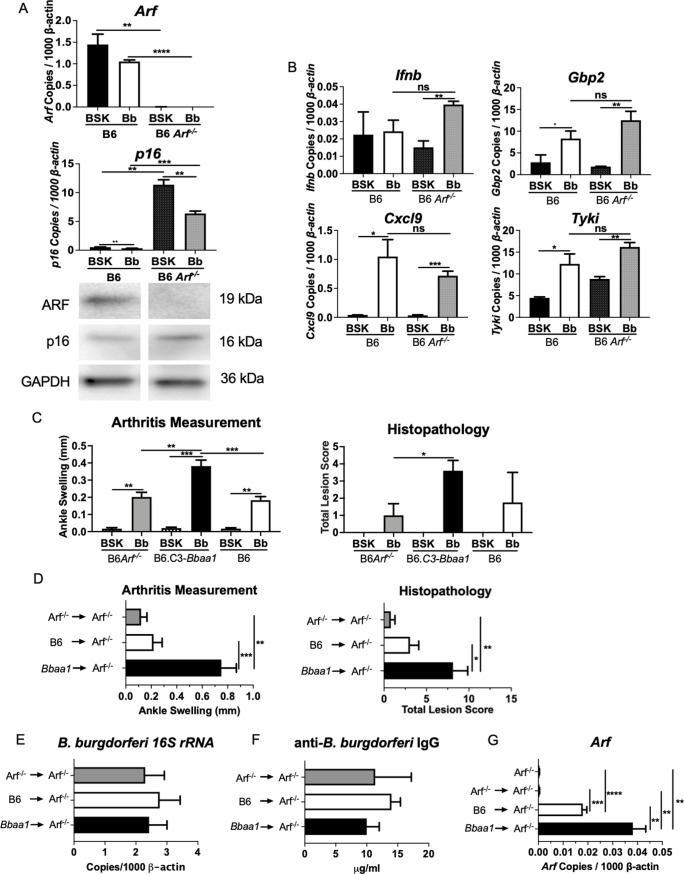
The C3H *Bbaa1* allele of ARF enhances arthritis severity in *B*. *burgdorferi*-infected mice. A) BMDMs isolated from B6 and B6 Arf^-/-^ mice were treated with sonicated *B*. *burgdorferi* for 6 h to induce the IFN response. The impact of ablation only on ARF expression, but not p16, was confirmed by qPCR and Western blot, and performed twice. Significance was determined by Student *t*-test. Error bars indicate SEM (n = 3 per group). B) Sonicated *B*. *burgdorferi*-stimulated *Ifnb*, *Gbp2*, *Tyki*, and *Cxcl9* expression levels in B6 Arf^-/-^ and B6 BMDMs were determined by qPCR normalized to *β-actin*. The *B*. *burgdorferi-*stimulated IFN response is compared with the BSK media alone group. Significance of differences was measured by Student *t*-test. Data are shown as mean ± SEM (n = 3 per group). C) B6 Arf^-/-^, B6.C3-*Bbaa1*, and B6 mice were infected with live *B*. *burgdorferi* at 6 or 7 weeks of age. Arthritis severity was measured by ankle measurement (ankle swelling) and by histopathologic assessment (total score) at 4 weeks following infection. There were *≥* 5 mice of mixed sex in each infected mouse group. Data were pooled from 2 separate experiments. Statistical analysis was assessed by Student *t*-test. Error bars indicate SEM. D) Reconstitution of B6 Arf^-/-^ mice with splenocytes from B6.C3-*Bbaa1* congenic mice or B6 mice revealed the contribution of the C3H *Bbaa1* allele of ARF to the development of severe Lyme arthritis. Mice were infected 3 weeks after reconstitution, and arthritis severity was measured at 4 weeks post-infection by ankle measurement (ankle swelling) and by histopathologic assessment (total score). The direction of transplantation from donor to recipient is shown on the figure. Results from two separate experiments with ≥ 5 mice in each infected mouse group were pooled. Statistical analysis was assessed by Student *t*-test. Error bars indicate SEM (n = 5 or 8 or 10 per group). E) *B*. *burgdorferi* in joint tissue was quantified by *16S rRNA* PCR (normalized to *β-actin*) and F) by ELISA quantification of serum anti-*B*. *burgdorferi* IgG. Significance was calculated by Student *t*-test. (n = 5 or 8 or 10 per group). G) The efficiency of ARF reconstitution in recipient B6 Arf^-/-^ mice was determined by qPCR of RNA isolated from whole blood. qPCR analysis was normalized to *β-actin*. Significance was calculated by Student *t*-test. Error bars indicate SEM (n = 5 or 8 or 10 per group). *p < 0.05, **p < 0.01, ***p < 0.001, ****p < 0.0001.

BMDMs from ARF-deficient B6 mice were used to test the impact of the null allele on the IFNβ response to *B*. *burgdorferi*. As expected, BMDMs from B6 Arf^-/-^ mice failed to express the *Arf* transcript and ARF protein (Fig 6A). Interestingly, BMDMs from B6 and B6 Arf^-/-^ mice expressed barely detectable levels of *Ifnb* and low levels of ISGs after *B*. *burgdorferi* stimulation suggesting that the B6 allele of ARF does not contribute appreciably to the *B*. *burgdorferi*-induced IFNβ response (Fig 6B). Arthritis severity was determined by ankle swelling and histopathological scores, and the arthritis observed in *B*. *burgdorferi*-infected B6 Arf^-/-^ mice was mild and similar to that observed in *B*. *burgdorferi*-infected B6 mice and significantly lower than that observed in *B*. *burgdorferi*-infected B6.C3-*Bbaa1* mice (Fig 6C). This indicates that the increased expression of ARF conferred by the C3H allele is responsible for the elevated IFNβ response in C3H and B6.C3-*Bbaa1* mice.

To test the impact of ARF reconstitution on IFNβ production and the Lyme arthritic response, radiation chimeras were generated by reconstituting B6 Arf^-/-^ mice with hematopoietic cells from B6.C3-*Bbaa1* or B6 mice. A rapid reconstitution protocol was used that allowed mice to be irradiated at 5 weeks of age and to achieve sufficient reconstitution by 8 weeks of age for infection with *B*. *burgdorferi*; the 8-week timepoint for infection is necessary for maximal arthritis development. We previously reported that B6.C3-*Bbaa1* mice reconstituted with B6.C3-*Bbaa1* splenocytes retained severe arthritis upon infection [27]. At 4 weeks post-infection, the B6 Arf^-/-^ recipient mice developed severe Lyme arthritis if they received splenocytes from B6.C3-*Bbaa1* mice, but not from B6 or B6 Arf^-/-^ mice (Fig 6D). The reconstitution had no effect on the host immune response as determined by the level of *Borrelia 16S rRNA* in joint tissue (Fig 6E) and the level of serum anti-*B*. *burgdorferi* IgG at 4 weeks post-infection (Fig 6F). The efficiency of ARF reconstitution in recipient B6 Arf^-/-^ mice was determined by PCR analysis of RNA isolated from whole blood (Fig 6G). These findings support the hypothesis that increased expression of ARF bestowed by the C3H allele is responsible for the upregulated IFNβ response that drives severe Lyme arthritis in C3H and B6.C3-*Bbaa1* mice. Interestingly, the B6 allele of ARF does not appear to play a role in the modest *B*. *burgdorferi*-induced IFNβ response in BMDMs or in the development of mild Lyme arthritis. Thus, the effect on IFNβ production is only manifest when the C3H gain of function allele of ARF is expressed.

### The involvement of p53 in ARF regulation of IFNβ

**Fig 7:**
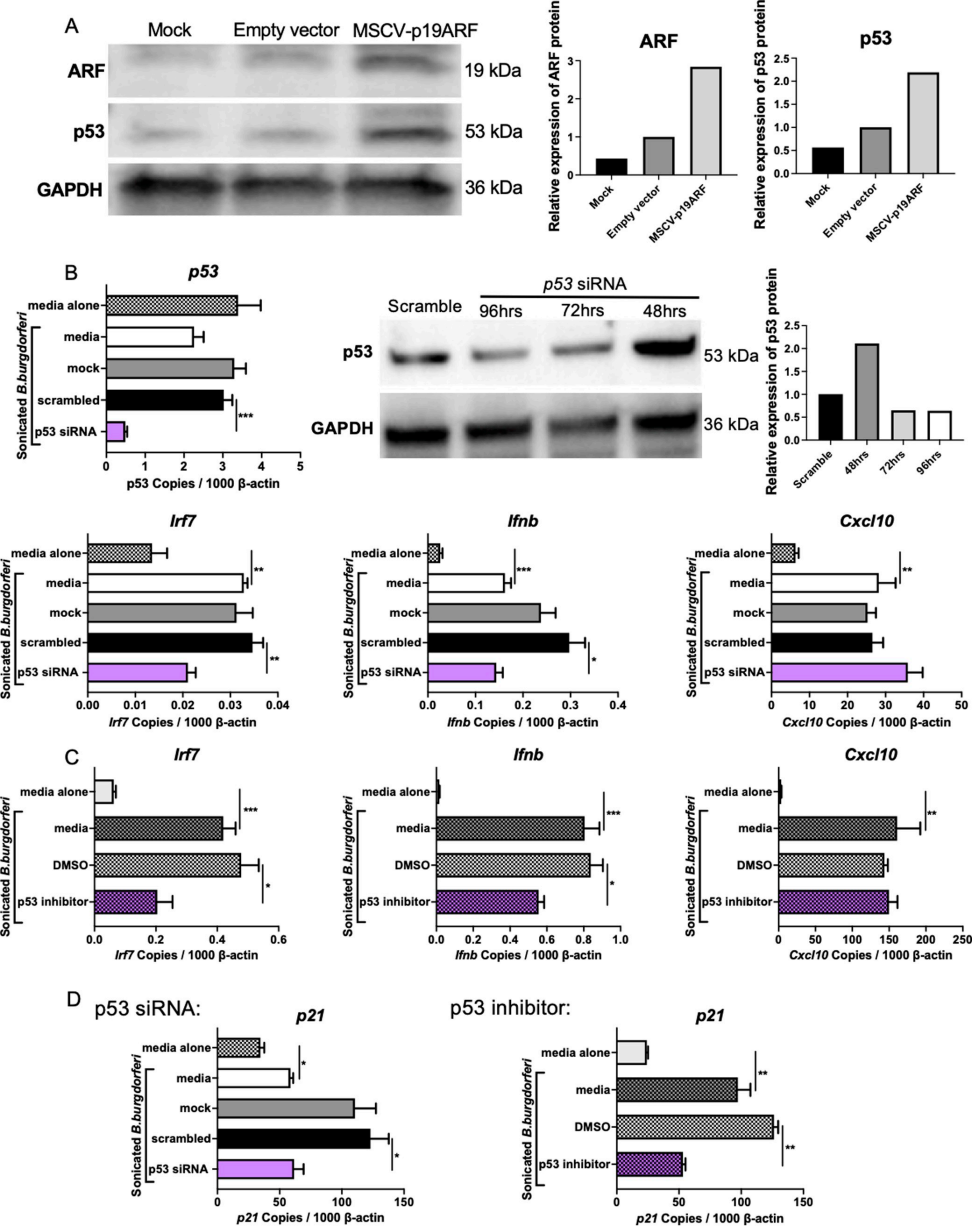
The involvement of p53 in IFNβ expression. A) ARF was overexpressed in B6 BMDMs by transfection of the MSCV-p19ARF plasmid and expression assessed by Western Blot. The transfected cells were allowed to rest for 72 h before the addition of sonicated *B*. *burgdorferi* for 6 h. B) p53 was silenced in B6.C3-*Bbaa1* BMDMs by transfection with 100 nM *p53* siRNA. Transfected cells were stimulated with sonicated *B*. *burgdorferi* prior to collection for RNA and protein extraction. IFN responses was induced by *B*. *burgdorferi* treatment as indicated by *B*. *burgdorferi* treated media, while samples receiving no stimulus are indicated as media alone. Cells were collected and protein was isolated at 48 h, 72 h, and 96 h to assess for silencing efficiency by Western Blot. *p53* transcripts were analyzed by qPCR normalized to *β-actin*. Significance was determined by Student *t*-test. Data are shown as mean ± SEM (n = 3 per group). The impact of *p53* silencing on expression of sonicated *B*. *burgdorferi*-stimulated IFN responses in B6.C3-*Bbaa1* BMDMs was determined by qPCR normalized to *β-actin*. Significance of differences was calculated by Student *t*-test. Data are shown as mean ± SEM (n = 3 per group). C) BMDMs were treated with PFTα for 1 h prior to stimulation with sonicated *B*. *burgdorferi* for 6 h. qPCR was used to assess the impact of the inhibitor on expression of *Ifnb* and downstream genes, and data were normalized to *β-actin*. Significance was determined by Student *t*-test. Data are shown as mean ± SEM (n = 3 per group). D) Expression of a p53 downstream gene, *p21*, was used to verify the efficiency of blocking p53 with siRNA and the PFTα inhibitor. Expression was measured by qPCR normalized to *β-actin*. Significance of difference was calculated by Student *t*-test (n = 3 per group). *p < 0.05, **p < 0.01, ***p < 0.001.

The canonical function of ARF is to bind and inactivate the p53 ubiquitin ligase MDM2 [46], thereby stabilizing p53 to function during cell cycle arrest (Fig 2A). Additionally, p53 was recently reported to bind and stabilize the *Irf7* and *Irf9* transcripts resulting in increased levels of IRF7 and IRF9 proteins and enhanced expression of IFNβ after viral infection [47]. Consistent with this alternative function of p53, BMDMs from ISRLC3 and ISRCL4 congenic mice expressed higher IRF7 transcript levels than BMDMs from wild type B6 mice (S3 Fig) suggesting that the C3H allele of ARF may lead to stabilization of the p53 protein, which would subsequently lead to increased expression of *Irf7* and *Irf9* transcripts and proteins [47]. Because IRF7 is an important transcription factor for IFNβ, we propose that the enhanced expression of the C3H allele of ARF leads to a p53-dependent increase in IRF7 mRNA and protein expression and a subsequent increase in IFNβ expression. In support of this hypothesis, overexpression of ARF resulted in increased levels of p53 (Fig 7A).

Silencing of p53 in B6.C3-*Bbaa1* BMDMs resulted in reduced expression of *Irf7* and *Ifnb*, but not *ISGs*; the reduced expression was evident by 48 h even though it took 72 h to observe reduced p53 expression (Fig 7B). Although *Irf7* and *Ifnb* transcripts were reduced upon silencing of p53, downstream ISGs were not suppressed as shown for *Cxcl10*. The incomplete silencing of p53 made it difficult to determine whether p53 is essential for IFNβ expression. A second approach was to interfere with p53 activity using the small molecule p53 inhibitor PFTα. PFTα blocks p53 function by decreasing the stability of nuclear p53 and disrupting its ability to regulate p53-responsive genes [48]. BMDMs from B6.C3-*Bbaa1* mice were plated overnight and 30 μΜ of PFTα was added to the cells 1 h before the addition of sonicated *B*. *burgdorferi*. Inhibition of p53 with PFTα resulted in reduced expression of *Irf7* and *Ifnb*; however, ISGs, such as *Cxcl10*, remained unaffected (Fig 7C). These results are consistent with the results of the p53 silencing experiment and suggest that p53 is important for IFNβ expression and early transcription of IRF7, but does not affect expression of ISGs. Another experiment utilized the cell cycle regulatory protein p21, which relies on p53 for expression via a series of intermediates distinct from those involved in IRF7 expression. RNA silencing and small molecule inhibition of p53 suppressed p21 expression only 50% and 60%, respectively (Fig 7D). These findings suggest that there is a technical limitation to silencing and inhibiting p53 on expression of downstream IFNβ and p21. In addition, the partial contribution of p53 to the IFNβ response suggests that other pathways may also play a role in ARF-modulated expression of IFNβ.

### The involvement of BCL6 in ARF regulation of IFNβ

**Fig 8:**
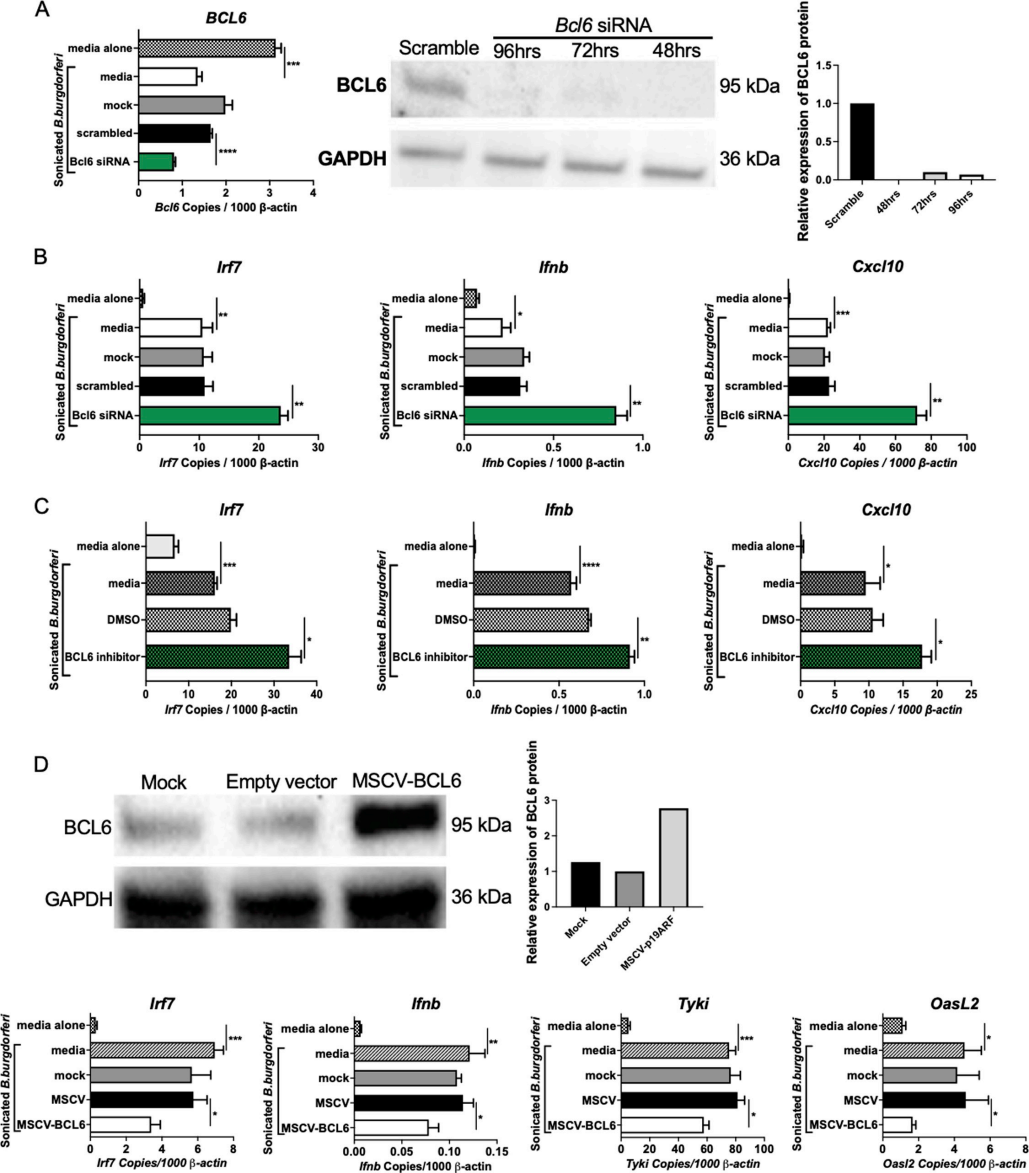
BCL6 plays a role in regulating IFNβ activity. A) *Bcl6* was silenced in B6.C3-*Bbaa1* BMDMs by transfection of *Bcl6* siRNA. Transfected cells were allowed to rest for 48 h and were then incubated with sonicated *B*. *burgdorferi* for 6 h prior to collection for RNA isolation. Transfected cells were incubated for 48 h, 72 h, and 96 h before treatment with sonicated *B*. *burgdorferi* for 6 h prior to collection for protein isolation. The induction of IFN responses is indicated by comparing *B*. *burgdorferi* treated samples (media) with unstimulated media alone. *Bcl6* expression was measured by qPCR normalized to *β-actin*. Significance was determined by Student *t*-test. Data are shown as mean ± SEM (n = 3 per group). B) The impact of *Bcl6* silencing on expression of *B*. *burgdorferi-*stimulated IFN responses in B6.C3-*Bbaa1* BMDMs was determined by qPCR normalized to *β-actin*. Significance of differences was determined by Student *t*-test. Data are shown as mean ± SEM (n = 3 per group). C) B6.C3-*Bbaa1* BMDMs were treated with 13 μM FX1 for 0.5 h before the addition of sonicated *B*. *burgdorferi* for 6 h. Blocking of BCL6 induced expression of *Irf7*, *Ifnb*, and ISGs, which was determined by qPCR normalized to *β-actin*. Significance of differences was calculated by Student *t*-test. Error bars indicate SEM (n = 3 per group). D) Overexpression of BCL6 by transfection of B6.C3-*Bbaa1* BMDMs with 1 μg MSCV-BCL6 plasmid was performed to confirm transcriptional repression by BCL6. Transfected cells were allowed to rest for 48 h and were then incubated with sonicated *B*. *burgdorferi* for 6 h prior to isolation of RNA and protein. The impact of BCL6 overexpression on activation of *Ifnb* and ISGs upon stimulation with *B*. *burgdorferi* in B6.C3-*Bbaa1* BMDMs was determined by qPCR normalized to *β-actin*. Significance was calculated by Student *t*-test. Error bars indicate SEM (n = 3 per group). *p < 0.05, **p < 0.01, ****p < 0.0001.

ARF is also known to bind and suppress the transcriptional repressor BCL6, which is expressed in T cells, B cells, and macrophages [49]. In myeloid cells, BCL6 has been shown to bind the *Irf7* promoter and suppress *Irf7* transcription resulting in reduced IFNβ expression [50]. Consistent with this, RNA-seq data showed increased constitutive and induced *Irf7* expression in BMDMs from congenic mice than in BMDMs from WT B6 mice **(**S3 Fig). Thus, ARF may modulate IFNβ expression through a second pathway involving BCL6-regulated expression of IRF7 in myeloid cells. Upon silencing of *Bcl6* expression in BMDMs from B6.C3-*Bbaa1* mice, the *Bcl6* transcript was reduced 60% and BCL6 protein was reduced 80% (Fig 8A). The silencing of *Bcl6* enhanced the *B*. *burgdorferi-*induced expression of *Irf7*, *Ifnb*, and ISGs, which is consistent with previous reports (Fig 8B). FX1 is a BCL6 inhibitor that binds to the BCL6 BTB domain and prevents direct interaction with corepressors required for BCL6 gene repression activity [51]. Treatment of B6.C3-*Bbaa1* BMDMs with FX1 resulted in increased expression of *Irf7*, *Ifnb*, and ISGs upon stimulation with *B*. *burgdorferi*; this increased expression was comparable to the expression achieved with siRNA silencing of BCL6 in B6.C3-*Bbaa1* BMDMs (Fig 8C). The direct impact of BCL6 on IFNβ expression was further studied by overexpressing BCL6 in BMDMs from B6.C3-*Bbaa1* mice. Transfection of the MSCV promoter-driven human *Bcl6* plasmid resulted in reduced expression of *Irf7*, *Ifnb*, and ISGs, whereas the empty MSCV vector had no effect on expression of these transcripts (Fig 8D). Taken together, these findings support the model in which ARF interacts with BCL6, thereby enhancing *Irf7* expression and the subsequent upregulation of IFNβ in myeloid cells. The C3H allele of ARF reduces the availability of BCL6 allowing activation of IRF7 and increased IFNβ production.

### A small molecule inhibitor of BCL6 modulates development of B. burgdorferi-induced Lyme arthritis

**Fig 9:**
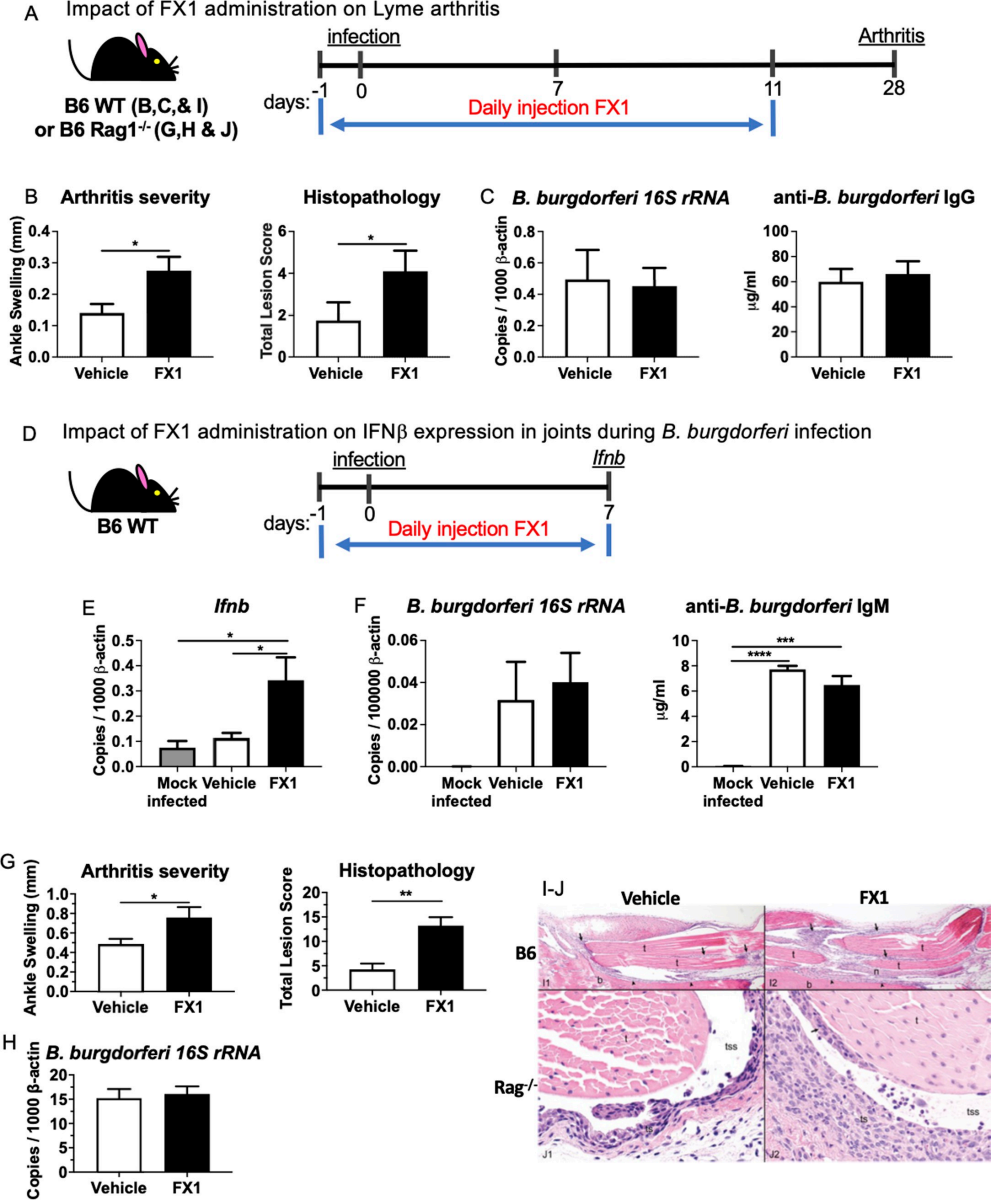
Suppression of BCL6 enhances induction of IFNβ and development of severe arthritis in joint tissues of B6 mice and B6 Rag1^-/-^ mice. A) B6 mice (6–7 weeks of age) that were infected with live *B*. *burgdorferi* on day 1 received daily i.p. injections of FX1 (50 mg/kg) starting the day before infection and continuing for 12 days. B) Arthritis was assessed at 28 days post-infection by measuring ankle swelling and by histopathologic determination (total score). Data were pooled from 2 separate experiments. Statistical analysis was performed using the Student *t*-test. Error bars indicate SEM (n = 9 or 10 per group). C) *B*. *burgdorferi* was quantified in joint tissues by *16S rRNA* PCR normalized to *β-actin* and by ELISA quantification of serum anti-*B*. *burgdorferi* IgG. Significance was calculated by Student *t*-test (n = 9 or 10 per group). D) The impact of blocking BCL6 on *Ifnb* expression was assessed at 1week post-infection in mice treated daily with 50 mg/kg FX1 beginning the day before infection and continuing until the mice were sacrificed at day 7 post-infection. E) RNA was isolated from joint tissues and *Ifnb* expression was measured by qPCR normalized to *β-actin*. Data were pooled from 2 separate experiments. Significance was calculated by Student *t*-test (n = 9 or 10 per group). F) Host defense was assessed by qPCR of *B*. *burgdorferi 16S rRNA* in joint tissues normalized to *β-actin* and by quantification of serum anti-*B*. *burgdorferi* IgM. Significance of differences was determined by Student *t*-test (n = 9 or 10 per group). G) *B*. *burgdorferi*-infected B6 Rag1^-/-^ mice (6–7 weeks of age) received daily i.p. injections of FX1 (50 mg/kg) starting the day before the infection and continuing for 12 days as described in Fig 7A. Arthritis was measured at 28 days post-infection and assessed by ankle swelling and histopathologic determination (total score). This experiment was repeated 3 times. Statistical analysis was performed using the Student *t*-test. Error bars indicate SEM (n = 5 per group). H) *B*. *burgdorferi* was quantified in joint tissue by *16S rRNA* PCR normalized to *β-actin*. Significance of difference was calculated by Student t-test (n = 5 per group). *p < 0.05, **p < 0.01, ***p < 0.001, ****p < 0.0001. (I-J) Responses of rear ankle joints (i.e., tibiotarsal and other connected joints) to injury in IFNβ mediated Lyme arthritis. I1) Vehicle treated infected B6 mice. Note minimal to mild thickening of the sheaths (arrows) of the tendons (t) and minimal to mild thickening of the periosteum (arrowheads) of the bone (b). Minimal numbers of neutrophils and/or mononuclear inflammatory cells are present in the tendon sheath spaces (not visible in the image). H&E stain. I2) FX1 treated infected B6 mice. Note moderate thickening of the tendon sheaths (arrows) and hypertrophy/hyperplasia of synoviocytes covering the tendons (t). Mild to moderate thickening of the periosteum (arrowheads) of the bone (b) is also present. Moderate numbers of neutrophils and mononuclear inflammatory cells are present in the tendon sheath spaces (not visible in the image). A nerve (n) is present in the specimen. H&E stain. J1) Vehicle treated infected B6 Rag^-/-^ mice. Note minimal to mild thickening (hypertrophy/hyperplasia) of the tendon sheath (ts) and minimal numbers of neutrophils and/or mononuclear inflammatory cells in the tendon sheath space (tss). H&E stain. J2) FX1 treated infected B6 Rag^-/-^ mice. Note moderate thickening of the tendon sheath (ts) and hypertrophy/hyperplasia of synoviocytes (arrow) covering the tendon (t). Moderate numbers of neutrophils and minimal numbers of mononuclear inflammatory cells are present in the tendon sheath space (tss). H&E stain.

Because treatment of BMDMs with the BCL6 inhibitor FX1 resulted in increased production of IFNβ and downstream ISGs, we hypothesized that treatment with FX1 may also enhance IFNβ production *in vivo* and alter the severity of Lyme arthritis. This hypothesis was tested in mildly arthritic B6 mice by administering FX1 or a vehicle control (30% propylene glycol, 5% Tween 80, and 65% D5W) daily by i.p. injection for 11 days beginning the day before infection with *B*. *burgdorferi* (Fig 9A). This treatment encompassed the previously determined day 7 peak of *Ifnb* expression in joint tissue (Fig 9A) [25,26]. Treatment of *B*. *burgdorferi*-infected B6 and B6 Rag1^-/-^ mice with the BCL6 inhibitor FX1 resulted in greater rear ankle swelling, and increased severity of scored parameters of arthritis such as thickening of the tendon sheaths and hypertrophy/hyperplasia of synoviocytes (Fig 9B, 9I and 9J) to the level seen in B6.C3-*Bbaa1* mice (Fig 6C). Importantly, the levels of serum anti-*B*. *burgdorferi* IgG indicated that the host’s adaptive immune response was not impaired by the FX1 treatment (Fig 9C). FX1-treated animals also displayed similar levels of *Borrelia 16S rRNA* in joint and other tissues as vehicle-treated control animals at 4 weeks post-infection (Fig 9C).

To determine if the increased level of Lyme arthritis in FX1-treated B6 mice was preceded by a joint-localized spike in IFNβ production at day 7 post-infection as previously reported for infected C3H mice, FX1 was administered for 7 days starting the day before infection (Fig 9D). Control and FX1-treated mice were sacrificed at 7 days post-infection and *Ifnb* levels were measured. Higher levels of *Ifnb* were found in joint tissues of FX1-treated mice than in vehicle-treated control mice (Fig 9E). The FX1 treatment did not affect host defenses as determined by similar levels of *B*. *burgdorferi 16S rRNA* in joints and serum anti-*B*. *burgdorferi* IgM levels in FX1-treated and vehicle-treated control animals (Fig 9F). These data suggested that treatment of B6 mice with the inhibitor FX1 results in increased production of IFNβ and increased Lyme arthritis severity, similar to that observed in B6.C3-*Bbaa1* mice.

Because BCL6 plays a dominant role in T cell and B cell responses, the effect of FX1 treatment on Lyme arthritis severity was evaluated in B6 Rag1^-/-^ mice to determine the contribution of T cells and B cells to IFNβ production and arthritis (Fig 9G, 9H and 9J). As with B6 mice, B6 Rag1^-/-^ mice were treated with FX1 for 11 days beginning the day before infection, and arthritis was assessed at 28 days post-infection (Fig 9A). FX1-treated B6 Rag1^-/-^ mice displayed more severe Lyme arthritis than vehicle-treated control B6 Rag1^-/-^ mice indicating that BCL6 expression in non-lymphocytes, such as myeloid cells, is critical for the increased arthritis severity observed upon FX1 treatment (Fig 9G and 9J). The FX1 treatment did not affect host defenses as mice treated with FX1 and mice treated with vehicle harbored the same level of *B*. *burgdorferi 16S rRNA* in joints (Fig 9H).

**Fig 10:**
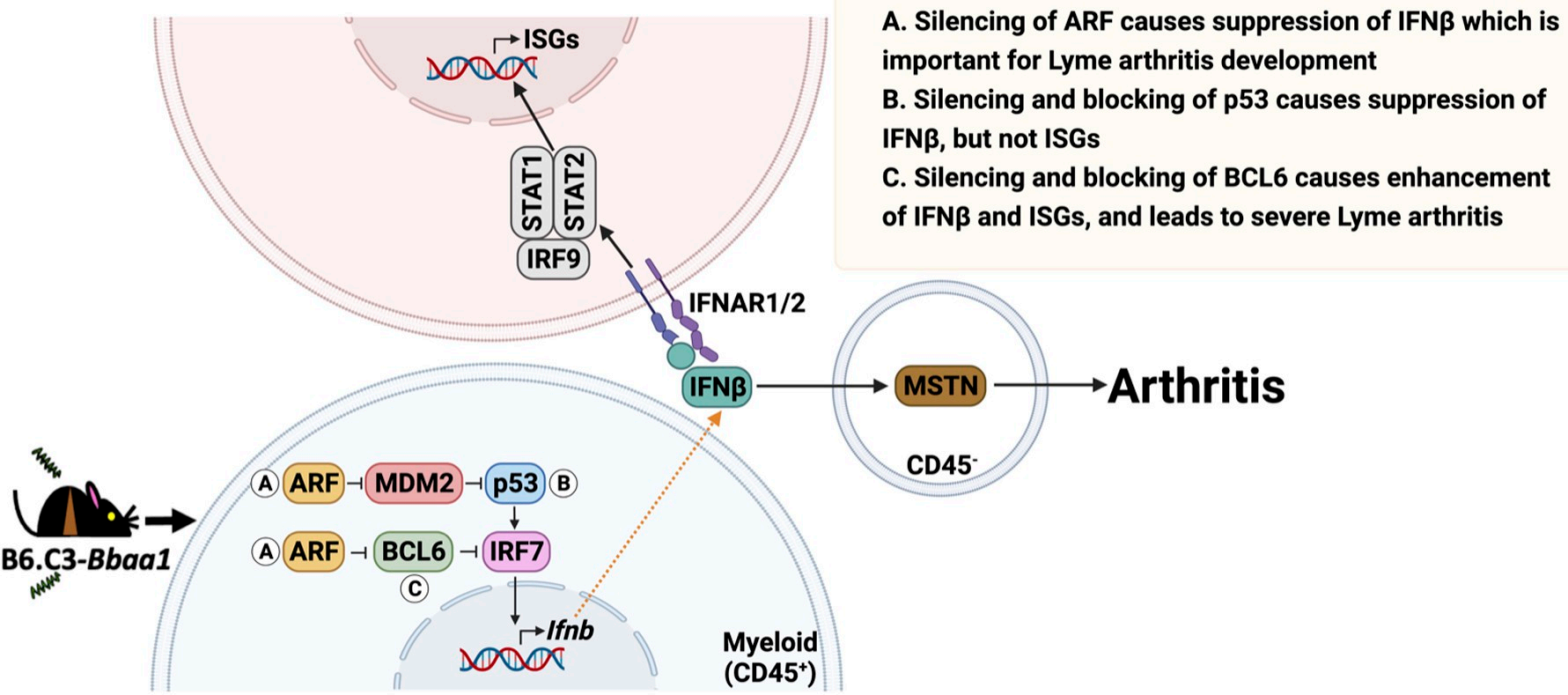
Proposed model of ARF-IFNβ pathway. A) The constitutively heightened expression of ARF in B6.C3-*Bbaa1* congenic mice results in a greater magnitude of IFNβ production and more severe Lyme arthritis following infection with *B*. *burgdorferi*. B) The heightened expression of the C3H allele of ARF blocks the activity of ubiquitin ligase MDM2, resulting in stabilized p53. p53 acts to enhance the expression of IRF7 which induces IFNβ production, but not downstream ISGs. C) The greater expression of the C3H allele of ARF also binds and suppresses BCL6, resulting in increased IRF7 expression and greater production of IFNβ. Expression of downstream ISGs is mediated through activation of signal transducer and activator transcription 1 (STAT1)-STAT2-IRF9 signaling complex. Parallel ARF interactions with p53 and BCL6 modulate IFNβ hyper production in resident myeloid cells. IFNβ leads to the upregulation of the muscle regulatory protein myostatin in CD45^-^ cells, a previously identified mediator of Lyme arthritis development in B6.C3-*Bbaa1* mice. (Created with BioRender.com).

Our *in vitro* mechanistic analysis of the *Cdkn2a*-encoded protein ARF demonstrated that ARF regulates IFNβ production via dual interaction with p53 and BCL6 in resident myeloid cells (CD45^+^) stimulated with *B*. *burgdorferi* (Fig 10). The *in vivo* experiments showed that the critical interactions occur in myeloid cells, not lymphoid cells, during the development of severe Lyme arthritis. By incorporating *in vitro* and *in vivo* data, we developed a model for the ARF-IFNβ pathway. Introgression of the C3H *Bbaa1* allele into B6 mice enhances expression of ARF, which blocks the p53 ubiquitin ligase MDM2 resulting in stabilized p53 and enhanced activation of IRF7 and production of IFNβ (Fig 10). The increased expression of ARF in congenic mice also blocks and inactivates BCL6 through protein-protein interactions, thereby increasing IRF7 production and upregulation of the IFNβ response. We previously demonstrated that IFNβ orchestrates Lyme arthritis by upregulating the production of the muscle regulatory protein myostatin (MSTN) by CD45^-^ resident cells of the joint [27] (Fig 10). These findings indicate potential sites of therapeutic intervention for IFNβ-modulated Lyme arthritis.

## Discussion

Genetic regulation of Lyme disease has been studied in humans and in inbred strains of mice [13–15]. Forward genetics using C3H and B6 mice, with varying Lyme arthritis severity, has allowed the development of congenic mice for study of genes involved in regulation of arthritis [20,21]. Previously, GUSB was positionally identified within B6.C3-*Bbaa2* on Chr5, and identified as a major determinant of Lyme arthritis severity in mice by regulating accumulation of pro-inflammatory lysosomal products in joint tissues [22,52]. A second approach using antibodies to block IFNβ protein and ablation of the type I IFN receptor 1 gene demonstrated that elevated production of IFNβ in myeloid lineage cells of joints is a major contributor to Lyme arthritis in C3H and B6.C3-*Bbaa1* mice [17,25,27]. In addition, elevated levels of type 1 IFN have been identified in samples from patients with Lyme disease and other IFN pathologies [30,53–56]. In this study, we used forward genetics to identify *Cdkn2a* as the gene within the *Bbaa1* locus on Chr4 that regulates IFNβ production in response to *B*. *burgdorferi* infection.

Interestingly, *Cdkn2a* is physically separated from the *Ifnb* gene on Chr4 by 0.75 Mb (Table 1 and Fig 2A) suggesting that *Cdkn2a* could be acting in cis to modulate IFNβ responses. However, an alternative hypothesis, that protein products of the *Cdkn2a* gene are working in trans to modulate IFNβ expression through intermediate factors, is supported by our findings. Of the two proteins encoded by *Cdkn2a*, ARF and p16 [28], only ARF regulates *Ifnb* expression in macrophages and Lyme arthritis severity in mice (Figs 2, 3, 4, 5 and 6D). This was supported by RNA-seq data (Fig 1C), by protein and RT-PCR quantification (S2 Fig), by silencing *Arf* in B6.C3-*Bbaa1* BMDMs (Fig 2B and 2C), and with radiation chimeras (Fig 6). Furthermore, the increased expression of the C3H allele of ARF upregulates IFNβ, which contributes to the development of severe Lyme arthritis in C3H and B6.C3-*Bbaa1* congenic mice. This is supported by overexpression of ARF in B6 BMDMs (Fig 5), by comparison of the *B*. *burgdorferi*-induced IFN response in B6 and B6 Arf^-/-^ BMDMs, and by comparison of *B*. *burgdorferi*-stimulated Lyme arthritis in B6 and B6 Arf^-/-^ mice (Fig 6B and 6C). Previously, Strle et al., demonstrated the role of TLR1 polymorphisms on Lyme disease severity in patients [13]. Interestingly, multiple SNPs in the human ARF gene (p14ARF) have been reported in endometrial cancer, possibly reflecting the important anti-tumor activity of p14ARF [57]. Our studies point to the possibility that SNPs in p14ARF could be linked to the severity of human Lyme disease.

The degree of IFNβ responses to a variety of PAMPs and another pathogen, *E*. *coli*, was influenced by expression of ARF, especially for PAMPs recognized by receptors utilizing the MyD88 adapter molecule (Table 2 and Fig 4B) [33,41–43]. However, IFNβ responses to the peptidoglycan subunit MDP and the dsRNA mimetic poly (I:C) were not reduced by ARF silencing (Table 2) indicating that NOD2 and TLR3 signaling are independent of ARF. The absence of ARF involvement in poly (I:C) induction of IFNβ suggests that the IFNβ response to at least some viral infections may be independent of ARF. Thus, ARF could serve as a potential therapeutic target for the treatment of Lyme disease without eliminating the defense against viral infections. In assessing similarities to other interferonopathies, a genome-wide association study has identified IFN-related genes in lupus, but not the *Cdkn2a* gene [58]. However, a recent study found *Cdkn2a* hypomethylation and expression alterations in systemic lupus erythematosus and systemic sclerosis [59] indicating that *Cdkn2a* regulation of IFN production is linked to additional syndromes. The strong link between type I IFN upregulation and lupus suggests that the ARF pathway could be involved in other pathologies.

p53 and BCL6 were previously identified as protein mediators of *Ifnb* production. ARF interacts with MDM2 stabilizing p53 protein, which in turn binds and increases IRF7 transcription [46,47], whereas BCL6 is a repressor of IRF7 transcription, which suppresses *Ifnb* expression [49,50]. RNA silencing and small molecular inhibitor studies provided evidence of p53 and BCL6 involvement in ARF-dependent regulation of IFNβ in BMDMs (Figs 7 and 8). These findings suggest that parallel ARF interactions with p53 and BCL6 modulate IFNβ production (Fig 10). Further, we demonstrated that B6 mice treated with the BCL6 inhibitor FX1 developed severe Lyme arthritis similar to B6.C3-*Bbaa1* mice (Fig 9). Infected B6 Rag^-/-^ mice treated with FX1 showed that development of severe Lyme arthritis did not involve lymphocytes (Fig 9G, 9H and 9J). In our model, we proposed that p53 and BCL6 function as parallel pathways. However, others have suggested that BCL6 interferes with p53 activity in chronic myeloid leukemia [60] indicating that BCL6 may partially act upstream of p53. Further investigation is required to understand the interactions between these two regulatory proteins in the regulation of microbial PAMPs, induction of IFNβ, and Lyme arthritis.

Although the primary role attributed to type I IFN is as a first line defense against viral infection, its upregulation has been observed in bacterial infection, where it can have both positive and negative impact on disease outcome [61]. In the case of *B*. *burgdorferi*, type I IFN mediates pathogenic arthritis development but not control of spirochete levels in tissues. Importantly, localized expression of type I IFN and ISGs has been reported in Lyme disease patients [30,35,62–64] and supports our finding of robust induction of type I IFN in joint tissues of mice with severe Lyme arthritis [23]. In addition, hepatitis C and multiple sclerosis patients treated with type I IFN may develop transient arthritis as a common side effect [65,66]. The pathologic role of type I IFN has also been observed in autoimmune diseases, such as systemic lupus erythematosus, Sjogren’s syndrome, and in a subgroup of rheumatoid arthritis patients [54,67–69]. Due to the multiple roles played by type I IFN, it is critical to discover its upstream and downstream mechanisms to provide more options for studying and treating type I IFN syndromes.

This mechanistic study positionally identified *Cdkn2a*-encoded ARF within the B6.C3-*Bbaa1* locus on Chr4 and demonstrated that ARF acts in trans to upregulate type I IFN through p53 and BCL6. Evidence for *Cdkn2a*-mediated regulation in hyper-IFN syndromes has been reported [59]. Our previous studies identified the muscle development regulatory protein MSTN as a major downstream effector of IFNβ induction and Lyme arthritis in mice [27]. Some patients with Lyme disease display fatigue and widespread musculoskeletal pain [70]; thus, the role of MSTN in Lyme disease patients remains to be investigated. Future studies will focus on identifying the therapeutic potential of upstream and downstream pathways of type I IFN and characterizing the downstream pathways of MSTN. These findings and future studies will allow assessment of new therapeutic targets for the treatment of type I IFN-dependent Lyme arthritis and provide support for the investigation of other IFN-driven diseases.

## Materials and methods

### Ethics statement

All mice used in this study were housed in the University of Utah Comparative Medicine Center and handled in strict accordance with the National Institutes of Health for the care and use of laboratory animals, as described in the Guide for the Care and Use of Laboratory Animals, 8^th^ Edition. All animal experiments were proved and performed according to the guidelines of the Institutional Animal Care and Use Committee (IACUC) at the University of Utah (Protocol Number 21–01002). Mouse experiments were conducted under isoflurane anesthesia, and every effort was made to minimize suffering.

### Mice

B6 mice were obtained from The Jackson Laboratory. B6.C3-*Bbaa1* congenic mice (Chr4: 11.6–93.46 Mbp) were generated by introgression of the *Bbaa1* allele from C3H onto the B6 background as described previously [20]. Continued backcrossing of B6.C3-*Bbaa1* mice with B6 mice allowed development of interval-specific recombinant congenic lines (ISRCL1-4) with the indicated Chr4 *Bbaa1* intervals: ISRCL 1 (11.6–77.8 Mbp), ISRCL2 (76.48–93.46 Mbp), ISRCL3 (83.7–93.46 Mbp), and ISRCL4 (88.3–93.46 Mbp) [20]. ISRCL5 (88.3–90.54 Mbp) was generated recently and the interval was fixed by filial mating as described previously [71]. C57BL/6 Arf^-/-^ (B6 Arf^-/-^) mice (B6.129X1-*Cdkn2a*^*tm1Cjs*^/KaiJ) were from Jackson laboratory, and were originally derived by Kamijo et al by disruption of Exon 1β [72]. This resulted in ablation of p19ARF, but not the p16 protein. C57BL/6 Rag1^-/-^ (B6 Rag1^-/-^) mice (B6.129S7-*Rag1*^*tm1/Mom*^/J) were obtained from The Jackson Laboratory and maintained on antibiotic water (trimethoprim and sulfamethoxazole), a treatment which does not impact on *B*. *burgdorferi*, prior to and during infection [73]. Mice were monitored daily for health status.

### B. burgdorferi infection of mice

The *B*. *burgdorferi* N40 isolate was provided by Dr. Stephen Barthold (University of California, Davis, CA) and was grown in Barbour-Stoenner-Kelly II (BSK) medium containing 6% rabbit serum (Sigma-Aldrich) [20]. Mice aged 6–8 weeks were infected with 2×10^4^ *B*. *burgdorferi* spirochetes by intradermal injection [74]. For mice that were sacrificed at 1-week post-infection, infection was confirmed by culturing *B*. *burgdorferi* from the bladder as described previously [75] and by measurement of *B*. *burgdorferi* specific IgM by ELISA. For mice that were sacrificed ≥ 2 weeks post-infection, infection was confirmed by the presence of *B*. *burgdorferi* specific IgM and IgG in serum measured by ELISA [75]. The presence of *B*. *burgdorferi 16S rRNA* transcripts in the joints was also used to confirm infection ≥ 7 days post-infection [23,76].

### Assessment of arthritis severity

Arthritis measurements were performed on rear ankle joints using a metric caliper on day 0 and day 28 post-infection by an individual blinded to the experimental group. The most severely swollen rear ankle was taken for histopathological analysis following removal of the skin. Joint tissue was fixed in 10% neutral buffered formalin, decalcified, embedded in paraffin, sectioned into 3 μm sections, and stained with hematoxylin and eosin (H&E) [77]. Lesions were scored blindly, and 6–8 sections per joint tissue were given a score of 0–5 based various aspects of disease, including severity and extent of the lesion, polymorphonuclear leukocyte and mononuclear cell (lymphocytes, monocyte, macrophage) infiltration, tendon sheath thickening (hypertrophy and hyperplasia of surface cells and/or underlying dense sheets of cells resembling immature fibroblasts, synoviocytes, and/or granulation tissue), and reactive/reparative responses (periosteal hyperplasia and new bone formation and remodeling). A score of 5 represents the most severe lesion and 0 represents no lesion as described previously [26].

### Generation of radiation chimeras

Chimeras were generated using a rapid reconstitution protocol that allowed infection within the 8-week window of maximal Lyme arthritis as described previously [25,73]. C57BL/6 Arf^-/-^ mice at 5 weeks of age were lethally irradiated with RS-2000 X-ray Biological Irradiator (Rad Source Technologies). Donor splenocytes (2×10^7^) harvested from B6.C3-*Bbaa1*, B6, and B6 Arf^-/-^ mice in 200 μl separation buffer (1X PBS pH 7.4, 2% FCS, 2 mM EDTA) were injected intravenously into irradiated B6 Arf^-/-^ recipient mice 24 h after irradiation. At 3 weeks post-irradiation and transplantation, recipient mice were infected with *B*. *burgdorferi*, and Lyme arthritis was assessed at 28 days post-infection. Chimerism was confirmed by PCR analysis of RNA isolated from whole blood at 4 weeks post-infection.

### Inhibitors and treatments

Inhibitors of p53 [Pifithrin-a (PFT-α)] and BCL6 (FX1) were obtained from Selleck Chemicals LLC. PFT-α (5 mM in DMSO) was stored at -80°C. For cell culture experiments, PFT-α was diluted to 30 μM with media containing 1% Nutridoma (Roche Applied Science). Cells were pretreated with PFT-α or DMSO for 1 h before the addition of stimuli [48]. For use in mice, PFT-α was diluted to 0.44 mg/ml with 30% propylene glycol, 5% Tween 80, and 65% D5W (5% dextrose dissolved in water). Mice (5–6/group) received daily intraperitoneal (i.p.) injections of 2.2 mg/kg PFT-α for 11 days beginning the day before *B*. *burgdorferi* infection [48]. The BCL6 inhibitor FX1 (40 mg/ml in DMSO) was stored at -80°C. For cell culture experiments, FX1 was diluted to 13 μM with 1% Nutridoma media and cells were pretreated with FX1 or DMSO for 0.5 h before the addition of stimuli [51]. For *in vivo* mouse studies, FX1 was diluted to 8 mg/ml with 30% propylene glycol, 5% Tween 80, and 65% D5W. Mice (5–6/group) were received daily i.p. injections of 50 mg/kg or 100 mg/kg FX1 for 11 days beginning the day before *B*. *burgdorferi* infection [51]. For both experiments, control mice received equivalent treatments with diluent. Drug administration was timed to span the peak of IFN signature transcripts in ankle tissue of *B*. *burgdorferi*-infected mice, at 7 days post-infection.

### Cell culture

Bone marrow-derived macrophages (BMDMs) were prepared by culturing bone marrow isolated from femurs and tibias of mice for 6 days in RPMI 1640 (Invitrogen Life Science) containing 30% L929 culture supernatant as the source of M-CSF and 20% horse serum (HyClone) at 37°C with 5% CO_2_ [78]. BMDMs were then harvested and replated into 24-well plates at a density of 6×10^5^ cells/ml in 0.5 ml of serum-free RPMI 1640 containing 1% Nutridoma and incubated overnight. The medium was removed and cells were stimulated for 6 h in 0.5 ml of 1% Nutridoma containing 5 μg/ml sonicated *B*. *burgdorferi*, which is approximately equivalent to 7.4×10^6^ live *B*. *burgdorferi*/ml, prepared as described previously and representing an approximate MOI of 10 [33]. The extraintestinal pathogenic *Escherichia coli* (ExPEC) strain, CFT073, was grown in 20 ml M9 minimal medium for 48 h at 37°C [29]. Following washing, *E coli* was added at 15 MOI relative to the macrophages [29,79]. *B*. *burgdorferi* RNA was prepared as described previously [33] and used at a dosage of 2 μg/ml. Polyinosinic-polycytidylic acid [poly (I:C); GE Healthcare Life Sciences] was used at 10 ng/ml, lipopolysaccharide (LPS; List Biological Laboratories) was used at 100 ng/ml, lipopeptide Pam_3_Cys-Ser-(Lys)4 (Pam3Cys;Abcam) was used at 100 ng/ml, and muramyl dipeptide (MDP; Sigma) was used at 10 μg/ml [33,42].

### Cell transfection

The Neon electroporation system (Life Technology) was used to transfect BMDMs with small interfering RNAs (siRNAs; Dharmacon) according to the manufacturer’s instructions. BMDMs were transfected with the indicated specific SMARTpool siRNAs, control scrambled SMARTpool siRNAs, or buffer. Transfected BMDMs were cultured in antibiotic-free RPMI 1640 medium (Invitrogen Life Technologies) containing 30% L929 culture supernatant and 20% horse serum (HyClone) in 48-well or 6-well plates and incubated at 37°C with 5% CO_2_. Cells were incubated in antibiotic-free medium for 48–72 h for recovery before the addition of stimulus in RPMI 1640 medium with 1% Nutridoma (Roche). Cells were incubated with stimuli for 6 h prior to extraction with TRIzol reagent for RNA analysis or with RIPA buffer for Western blot analysis. Efficiency of siRNAs transfection was determined by the reduction in detectable transcripts and proteins.

BMDMs were transfected with murine stem cell virus (MSCV)-p19ARF, MSCV-BCL6, or the MSCV empty vector (Addgene) at 1 μg/5×10^6^ cells/ml according to the manufacturer’s instructions. Transfected BMDMs were incubated in antibiotic-free RPMI 1640 medium in 24-well or 6-well plates for 72 h for recovery. The transfection efficiency of the plasmids was estimated at 80% by microscopy of GFP co-expression from each plasmid. The medium was then replaced with 1% Nutridoma containing the stimulus, and 6 h post-stimulation, cellular RNA or protein was extracted for qPCR or Western blot analysis, respectively.

### PCR

Total RNA was isolated with TRIzol reagent (Invitrogen) and purified using the Direct-zol RNA MiniPrep kit (Zymo Research). RNA was then reverse transcribed, and transcripts were quantified using a Roche LC-480 as described previously [20]. Primer sequences for *β-actin*, *Iigp* [23], *Oasl2*, *Cxcl10*, *Tyki*, *16S rRNA* [17], *Gbp2* [33], *Tnfa*, and *Ifnb* [80] were described previously. The other primer sequences were *Arf* forward: 5’-AAGAGAGGGTTTTCTTGGTG -3′ and *Arf* reverse: 5′- CATCATCATCACCTGGTCC -3′, *P16* forward: 5′- GCTGCAGACAGACTGGCC -3′ and *p16* reverse: 5′- CCATCATCATCACCTGAATCG -3′, *p53* forward: 5′- CTCTCCCCCGCAAAAGAAAAA -3′ and *p53* reverse: 5′- TAAACGCTTCGAGATGTTCCG -3′, and *Bcl6* forward: 5′-CCGGCACGCTAGTGATGTT-3′ and *Bcl6* reverse: 5’-TGTCTTATGGGCTCTAAACTGCT-3’.

### Western blot

Primary antibodies to BCL6 and GAPDH were purchased from Cell Signaling Technology. Primary antibodies to ARF and p53 were purchased from Abcam. Primary antibody to p16 was purchased from Santa Cruz Biotechnology, Inc. Secondary antibodies were purchased from Bio-Rad Laboratories (rabbit) and Invitrogen (mouse). Western blot analysis was performed following the general protocol from Abcam. Fiji-ImageJ was used to quantify Western blot data.

### Statistics

Statistical analysis was performed using Prism 8.0b software. Two sample data sets were analyzed using the Student *t*-test. Categorical variables were assessed by the Mann–Whitney U test. One-way analysis of variance (ANOVA) with the Tukey-Kramer multiple-comparison test was used for multigroup comparisons. Statistical significance was indicated as follows: *p < 0.05, **p < 0.01, ***p < 0.001, and ****p < 0.0001.

### RNA sequencing analysis

After RNA from B6, ISRCL3, and ISRCL4 BMDMs was extracted and purified with the Direct-zol RNA MiniPrep kit (Zymo Research), libraries were prepared using polyA enrichment and sequenced at the University of Utah High-Throughput Genomics Core Facility with the Agilent High Sensitivity RNA ScreenTape Assay as described previously [27]. Sequences were analyzed with help from the University of Utah Bioinformatics Analysis Core Facility as described previously [81–83]. The RNA-seq data shown in S1 Table have been submitted to the Gene Expression Omnibus repository under accession number GSE181071 at the National Center for Biotechnology Information (NCBI).

## Supporting information

S1 Fig Genetic factors in the 2 Mbp *Bbaa1* locus regulate differential expression of IFNβ and Lyme arthritis severity. A) At 7 days post-infection with live *B*. *burgdorferi*, a robust induction of IFNβ, which is regulated by the *Bbaa1* congenic region and leads to severe Lyme arthritis, was detected in joint tissues from B6.C3-*Bbaa1* mice. B) Further backcrossing reduced the physical interval of *Bbaa1* (left panel). The C3H allele of IFN and flanking genes were found to be required for development of Lyme arthritis (right panel). C3H-derived regions are colored black and B6-derived regions are colored white. Ankle swelling was measured at 4 weeks post-*B*. *burgdorferi* infection. Error bars indicate SEM (n = 10 to 35 mice per group). Significance was calculated by 1-way ANOVA followed by Dunnett’s multiple comparison test versus B6. ****p < 0.0001.
(TIF)

S2 Fig ARF expression is higher in congenic mice. A) Proteins were isolated from BMDMs from B6 and B6.C3-*Bbaa1* mice. The expression level of ARF protein was determined by western blot. B) BMDMs isolated from B6 and B6.C3-*Bbaa1* mice were treated with sonicated *B*. *burgdorferi* for 6 h to induce the IFN response. The *B*. *burgdorferi-*stimulated IFN response was compared between *B*. *burgdorferi* -treated group and media alone group. The *Arf* and *p16* expression levels were determined by qPCR normalized to *β-actin*. Significance was determined by Student *t*-test. Error bars indicate SEM (n = 3 per group). *p < 0.05, **p < 0.01.
(TIF)

S3 Fig IRF7 expression is higher in ISRCL3 and ISRCL4 mice. RNA-seq revealed higher constitutive and induced *Irf7* expression in BMDMs from ISRCL3 and ISRCL4 mice than in BMDMs from B6 mice following stimulation with live *B*. *burgdorferi* for 3 and 6 h. Error bars indicate SEM (n = 3 or 4 per group), *p < 0.05, **p < 0.01.
(TIF)

S1 Table RNA-seq identified all the genes within the *ISRCL5* interval. (DOCX)
