# Supplementary figures and images for: The Cdkn2a gene product p19 alternative reading frame (p19ARF) is a critical regulator of IFNβ-mediated Lyme arthritis
Source: PLoS Pathog. 2022 Mar 24;18(3):e1010365. doi: 10.1371/journal.ppat.1010365 (PMC8946740; doi:10.1371/journal.ppat.1010365)

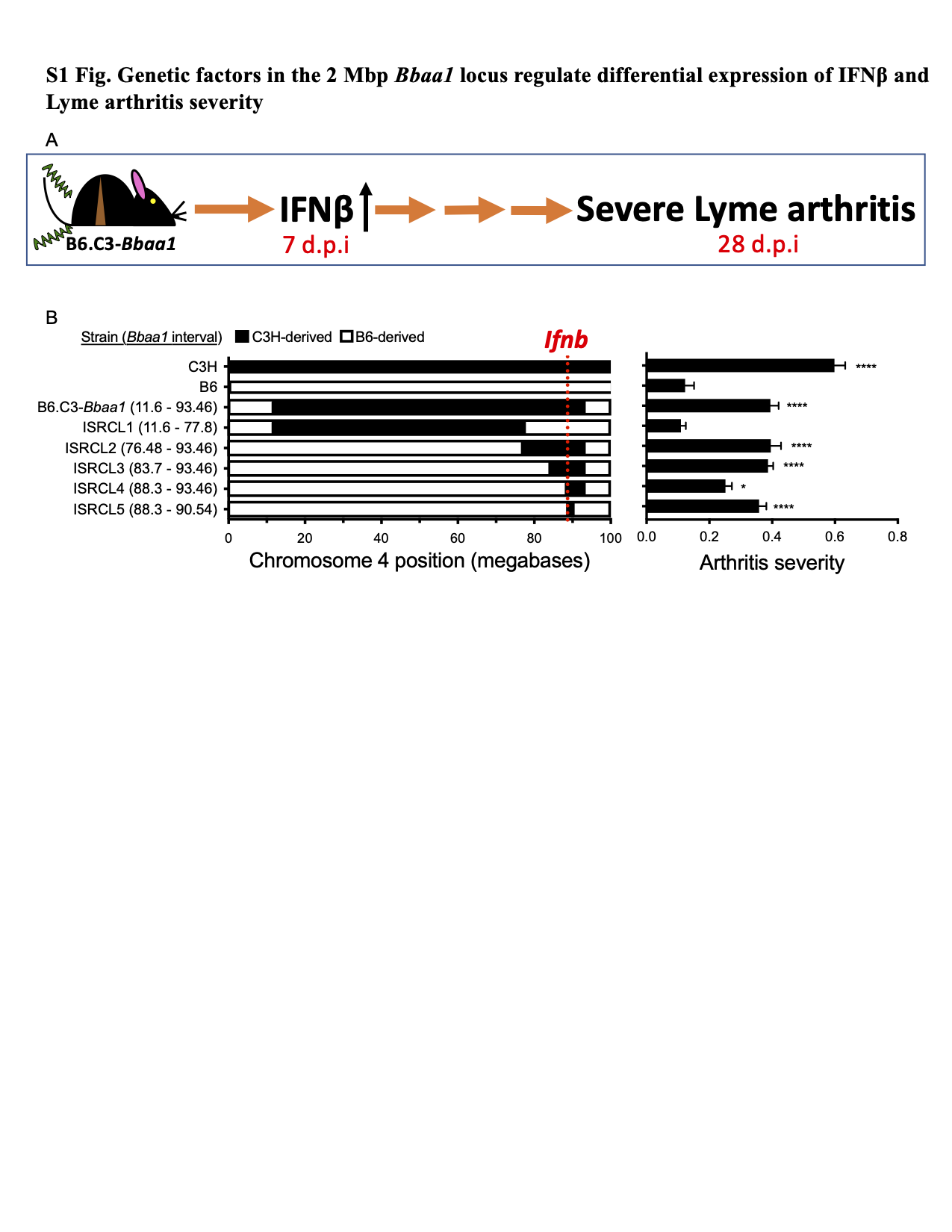

Supplement: S1 Fig — A) At 7 days post-infection with live B. burgdorferi, a robust induction of IFNβ, which is regulated by the Bbaa1 congenic region and leads to severe Lyme arthritis, was detected in joint tissues from B6.C3-Bbaa1 mice. B) Further backcrossing reduced the physical interval of Bbaa1 (left panel). The C3H allele of IFN and flanking genes were found to be required for development of Lyme arthritis (right panel). C3H-derived regions are colored black and B6-derived regions are colored white. Ankle swelling was measured at 4 weeks post-B. burgdorferi infection. Error bars indicate SEM (n = 10 to 35 mice per group). Significance was calculated by 1-way ANOVA followed by Dunnett’s multiple comparison test versus B6. ****p < 0.0001. (TIF) [file ppat.1010365.s001.tif]

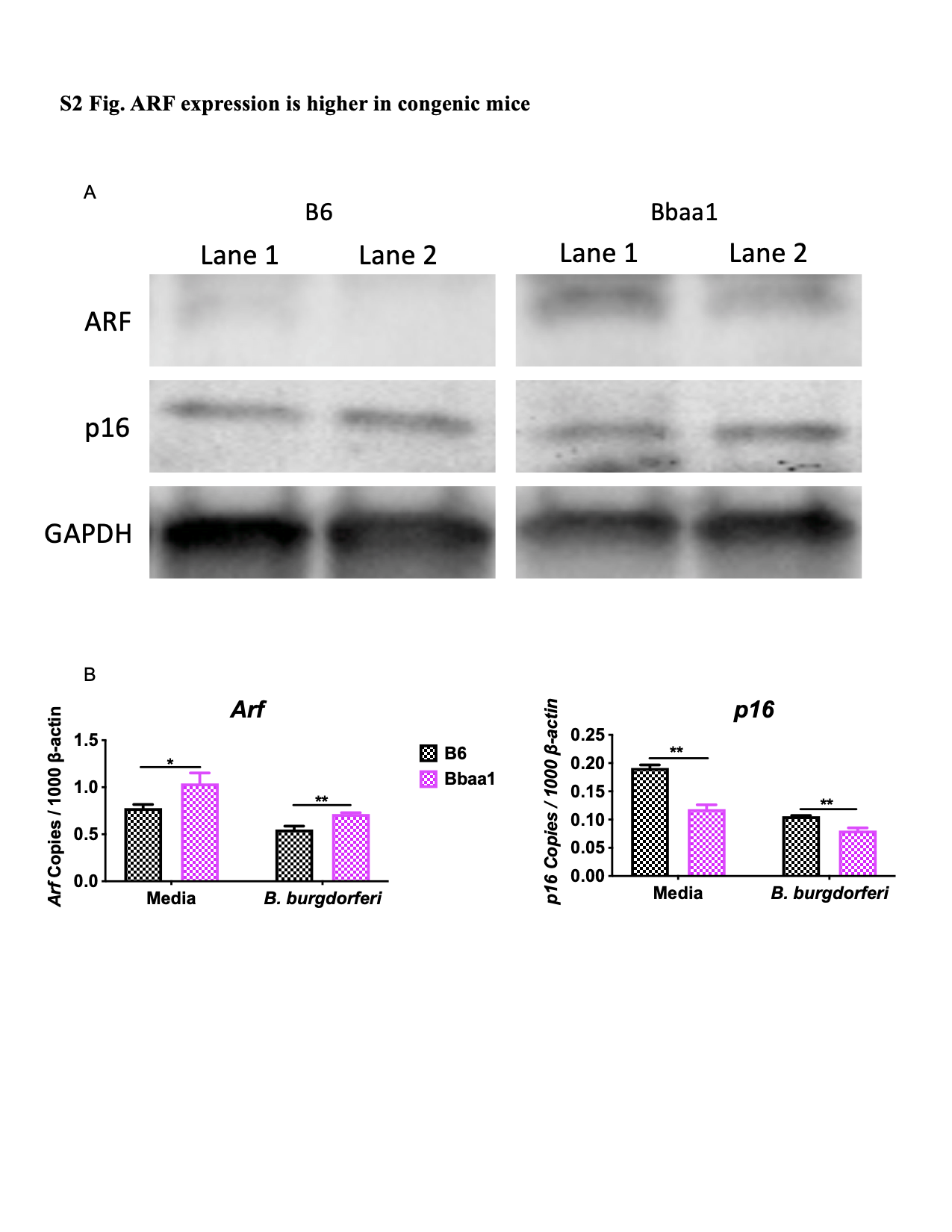

Supplement: S2 Fig — A) Proteins were isolated from BMDMs from B6 and B6.C3-Bbaa1 mice. The expression level of ARF protein was determined by western blot. B) BMDMs isolated from B6 and B6.C3-Bbaa1 mice were treated with sonicated B. burgdorferi for 6 h to induce the IFN response. The B. burgdorferi-stimulated IFN response was compared between B. burgdorferi -treated group and media alone group. The Arf and p16 expression levels were determined by qPCR normalized to β-actin. Significance was determined by Student t-test. Error bars indicate SEM (n = 3 per group). *p < 0.05, **p < 0.01. (TIF) [file ppat.1010365.s002.tif]

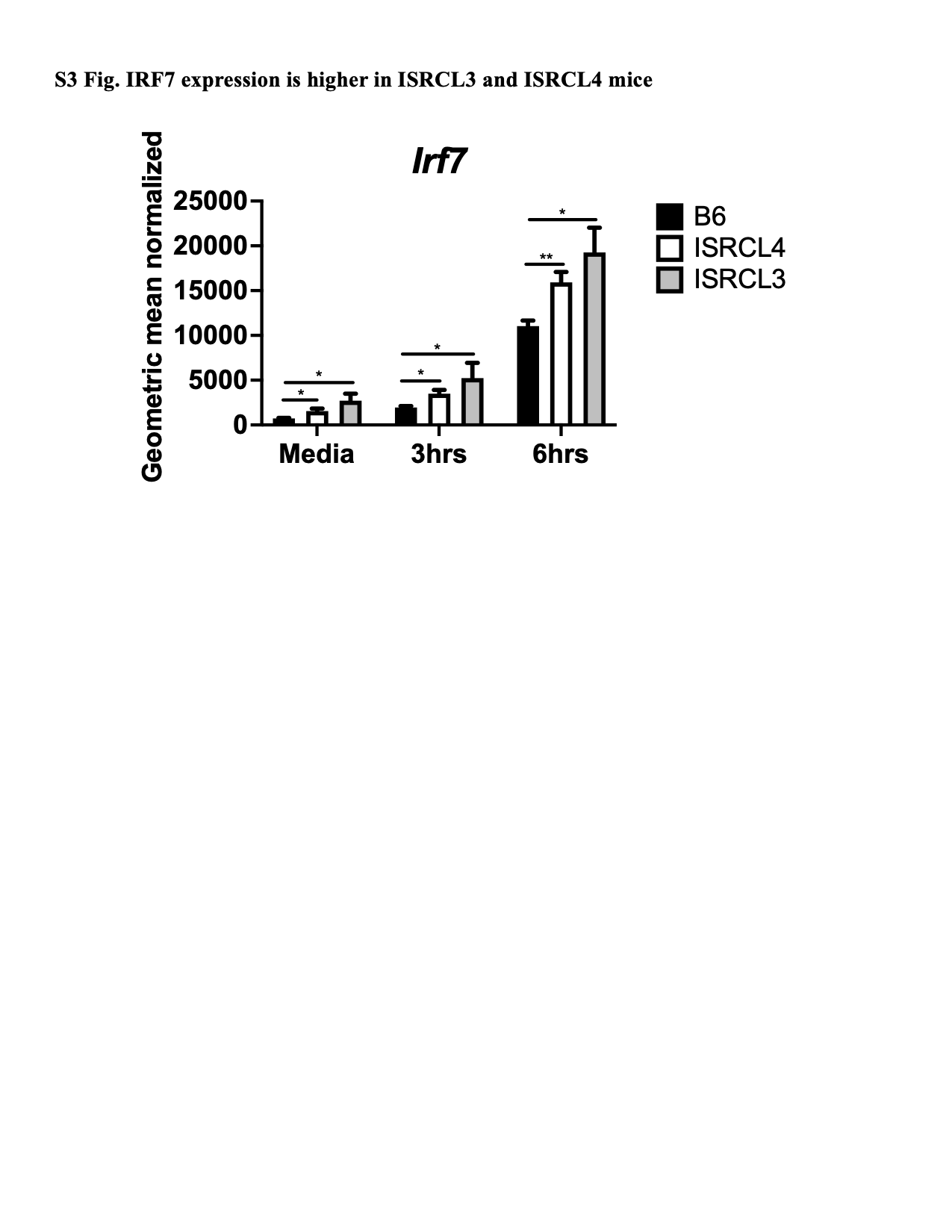

Supplement: S3 Fig — RNA-seq revealed higher constitutive and induced Irf7 expression in BMDMs from ISRCL3 and ISRCL4 mice than in BMDMs from B6 mice following stimulation with live B. burgdorferi for 3 and 6 h. Error bars indicate SEM (n = 3 or 4 per group), *p < 0.05, **p < 0.01. (TIF) [file ppat.1010365.s003.tif]
