# Supplementary material for: The Cdkn2a gene product p19 alternative reading frame (p19ARF) is a critical regulator of IFNβ-mediated Lyme arthritis
Source: PLoS Pathog. 2022 Mar 24;18(3):e1010365. doi: 10.1371/journal.ppat.1010365 (PMC8946740; doi:10.1371/journal.ppat.1010365)
Supplement: S1 Table — (DOCX) [file ppat.1010365.s004.docx]

**S1 Table. RNA-seq identified all the genes within the *ISRCL5* interval**.

| Gene symbol | Biotype | Chr4 position |
| --- | --- | --- |
| Focad | Protein coding | 4:88094629-88411011 |
| Mir491 | miRNA | 4:88122040-88122125 |
| Gm12645 | Processed pseudogene | 4:88151696-88151970 |
| Hacd4 | Protein coding | 4:88396144-88438928 |
| Ifnb1 | Protein coding | 4:88522025-88522774 |
| Ifna15 | Protein coding | 4:88557673-88558245 |
| Ifna14 | Protein coding | 4:88571229-88571798 |
| Ifna-ps1 | Unprocessed pseudogene | 4:88578887-88580574 |
| Ifna9 | Protein coding | 4:88591813-88592385 |
| Mrpl48-ps | Processed pseudogene | 4:88599175-88599810 |
| Ifna12 | Protein coding | 4:88602580-88603376 |
| Gm12601 | Unprocessed pseudogene | 4:88612653-88614044 |
| C87499 | Protein coding | 4:88627320-88634411 |
| Ifna13 | Protein coding | 4:88643641-88644459 |
| Gm13281 | Unprocessed pseudogene | 4:88648738-88649304 |
| Ifna16 | Protein coding | 4:88675915-88676924 |
| Ifna2 | Protein coding | 4:88682881-88683912 |
| Ifnab | Protein coding | 4:88690302-88691359 |
| Gm13273 | Unprocessed pseudogene | 4:88696191-88696698 |
| Gm13282 | Unprocessed pseudogene | 4:88707909-88708453 |
| Klhl9 | Protein coding | 4:88718292-88722465 |
| Gm26566 | Protein coding | 4:88721805-88722842 |
| Gm13274 | antisense | 4:88738838-88744294 |
| Gm13271 | Protein coding | 4:88754868-88755416 |
| Gm16686 | Protein coding | 4:88755195-88755590 |
| Gm13286 | Unprocessed pseudogene | 4:88757844-88758380 |
| Gm10583 | pseudogene | 4:88758171-88758570 |
| Gm13283 | Protein coding | 4:88760119-88761581 |
| Gm10582 | pseudogene | 4:88761101-88761500 |
| Gm13284 | Unprocessed pseudogene | 4:88763709-88771606 |
| Gm10581 | pseudogene | 4:88771550-88771784 |
| Gm13290 | Protein coding | 4:88773834-88774801 |
| Gm10580 | pseudogene | 4:88774321-88774720 |
| Gm13289 | Protein coding | 4:88776922-88777470 |
| Gm13272 | Protein coding | 4:88779850-88780660 |
| Ifnz | Protein coding | 4:88782131-88783592 |
| Gm26525 | lincRNA | 4:88784455-88794391 |
| Gm13276 | Protein coding | 4:88785709-88786257 |
| Gm13277 | Protein coding | 4:88788632-88789180 |
| Gm13278 | Protein coding | 4:88791550-88792098 |
| Gm13275 | Protein coding | 4:88794487-88795035 |
| Gm26867 | lincRNA | 4:88796143-88800224 |
| Gm13279 | Protein coding | 4:88797397-88797945 |
| Gm13285 | Protein coding | 4:88799665-88801126 |
| Gm13287 | Protein coding | 4:88803254-88803802 |
| Gm13288 | Protein coding | 4:88805531-88808380 |
| Ifna7 | Protein coding | 4:88816228-88816800 |
| Ifna11 | Protein coding | 4:88819959-88820531 |
| Ifna6 | Protein coding | 4:88827416-88827985 |
| Ifna5 | Protein coding | 4:88835525-88836094 |
| Ifna4 | Protein coding | 4:88841861-88842421 |
| Ifna1 | Protein coding | 4:88850087-88850656 |
| 4930553M12Rik | Protein coding | 4:88867882-88868379 |
| Ifne | Protein coding | 4:88879538-88880201 |
| Mir31 | miRNA | 4:88910557-88910662 |
| Gm12603 | lincRNA | 4:89050308-89084614 |
| Gm12602 | Processed transcript | 4:89100664-89128226 |
| Mtap | Protein coding | 4:89137122-89181081 |
| Gm12607 | Processed pseudogene | 4:89161699-89162191 |
| Gm26490 | snoRNA | 4:89182981-89183120 |
| Tgif2-ps1 | Processed pseudogene | 4:89216864-89217816 |
| Gm12606 | lincRNA | 4:89235699-89273403 |
| Cdkn2a | Protein coding | 4:89274471-89294653 |
| Cdkn2b | Protein coding | 4:89306289-89311032 |
| Gm12609 | Processed transcript | 4:89381957-89399880 |
| Gm12610 | Processed transcript | 4:89421848-89463487 |
| Gm12608 | Processed pseudogene | 4:89442217-89444644 |
| Gm27580 | Misc RNA | 4:89445219-89445367 |
| Dmrta1 | Protein coding | 4:89679436-89694772 |
| Gm12629 | lincRNA | 4:90185527-90190009 |
| Zfp352 | Protein coding | 4:90218820-90225702 |
| Gm12633 | Processed pseudogene | 4:90359622-90361326 |
| Gm12634 | Processed pseudogene | 4:90368731-90369722 |
| Gm12635 | Processed pseudogene | 4:90452874-90453625 |
| Gm12636 | Processed pseudogene | 4:90543747-90544370 |
| Gm12632 | Processed pseudogene | 4:90856883-90857377 |
| Gm25244 | snoRNA | 4:90997862-90997993 |
| Gm12643 | Processed pseudogene | 4:91037900-91038273 |
| Gm12644 | Processed pseudogene | 4:91156682-91157287 |
| Elavl2 | Protein coding | 4:91250763-91400785 |

RNA was prepared from ISRCL3, ISRCL4, and B6 BMDMs following stimulation with live *B. burgdorferi* for 3 h, 6 h, or without *B. burgdorferi* stimulation. *ISRCL5* is from 88.3–90.54 Mbp. The green areas indicate the region flanking *ISRCL5*.

B6: n = 4, ISRCL4: n = 4, ISRCL3: n = 3
